# Large-scale open-source three-dimensional growth curves for clinical facial assessment and objective description of facial dysmorphism

**DOI:** 10.1038/s41598-021-91465-z

**Published:** 2021-06-09

**Authors:** Harold S. Matthews, Richard L. Palmer, Gareth S. Baynam, Oliver W. Quarrell, Ophir D. Klein, Richard A. Spritz, Raoul C. Hennekam, Susan Walsh, Mark Shriver, Seth M. Weinberg, Benedikt Hallgrimsson, Peter Hammond, Anthony J. Penington, Hilde Peeters, Peter D. Claes

**Affiliations:** 1 Department of Human Genetics, KU Leuven, 3000 Leuven, Belgium; 2 Medical Imaging Research Center, UZ Leuven, Herestraat 49, 3000 Leuven, Belgium; 3 Facial Sciences Research Group, Murdoch Children’s Research Institute, Parkville, 3052 Australia; 4 School of Earth and Planetary Sciences, Faculty of Science and Engineering, Curtin University, Perth, 6845 Australia; 5 Western Australian Register of Developmental Anomalies, King Edward Memorial Hospital, Perth, Australia; 6 Telethon Kids Institute and Division of Paediatrics, Faculty of Health and Medical Sciences, University of Western Australia, Perth, Australia; 7 Faculty of Medicine, Notre Dame University, Fremantle, Australia; 8 Dept Clinical Genetics, Sheffield Children’s NHS Trust, OPDII Northern General Hospital, Herries Road, Sheffield, S5 7AU UK; 9 Program in Craniofacial Biology, Departments of Orofacial Sciences and Pediatrics, and Institute for Human Genetics, University of California, San Francisco, San Francisco, CA USA; 10 Human Medical Genetics and Genomics Program, University of Colorado School of Medicine, Aurora, CO USA; 11 Department of Pediatrics, Amsterdam University Medical Center, University of Amsterdam, Amsterdam, The Netherlands; 12 Department of Biology, Indiana University Purdue University Indianapolis, Indianapolis, IN 46202 USA; 13 Department of Anthropology, Pennsylvania State University, State College, PA 16802 USA; 14 Center for Craniofacial and Dental Genetics, University of Pittsburgh, Pittsburgh, PA 15219 USA; 15 Department of Cell Biology & Anatomy, Cumming School of Medicine, Alberta Children’s Hospital Research Institute, University of Calgary, Calgary, AB T2T 4N1 Canada; 16 Department of Plastic and Maxillofacial Surgery, Royal Children’s Hospital, Melbourne, 3052 Australia; 17 Department of Pediatrics, University of Melbourne, Melbourne, 3052 Australia; 18 Department of Electrical Engineering, ESAT/PSI, KU Leuven, 3000 Leuven, Belgium

**Keywords:** Diagnostic markers, Biomedical engineering, Statistical methods, Software

## Abstract

Craniofacial dysmorphism is associated with thousands of genetic and environmental disorders. Delineation of salient facial characteristics can guide clinicians towards a correct clinical diagnosis and understanding the pathogenesis of the disorder. Abnormal facial shape might require craniofacial surgical intervention, with the restoration of normal shape an important surgical outcome. Facial anthropometric growth curves or standards of single inter-landmark measurements have traditionally supported assessments of normal and abnormal facial shape, for both clinical and research applications. However, these fail to capture the full complexity of facial shape. With the increasing availability of 3D photographs, methods of assessment that take advantage of the rich information contained in such images are needed. In this article we derive and present open-source three-dimensional (3D) growth curves of the human face. These are sequences of age and sex-specific expected 3D facial shapes and statistical models of the variation around the expected shape, derived from 5443 3D images. We demonstrate the use of these growth curves for assessing patients and show that they identify normal and abnormal facial morphology independent from age-specific facial features. 3D growth curves can facilitate use of state-of-the-art 3D facial shape assessment by the broader clinical and biomedical research community. This advance in phenotype description will support clinical diagnosis and the understanding of disease pathogenesis including genotype–phenotype relations.

## Introduction

Craniofacial dysmorphism is a feature of thousands of genetic and environmental developmental disorders. Many genetic disorders present with a typical facial appearance that is readily identifiable. For others, the presentation may be more subtle; heterogeneous within, or atypical of, the disorder; shared among numerous disorders; or developing more fully over time. In the genomic era, delineation of salient facial characteristics is increasingly important for both clinical assessment and variant interpretation in whole-exome or whole-genome sequencing, thereby supporting molecular diagnosis. Further, abnormal facial shape is often an important indication for craniofacial interventions, with restoration of normal facial shape an important treatment outcome. In general, facial phenotypic analyses have relied primarily on linear measurements or facial gestalt recognition by trained dysmorphologists. Recent developments in bioinformatics, image processing and engineering now pave the way for more comprehensive, holistic and objective assessments of the facial phenotype. However, data and software for such assessments are frequently kept in-house, restricting broad implementation in clinics and research. This article addresses this shortcoming by deriving and presenting open-source models and code of ‘3D growth curves’ for the objective assessment of craniofacial dysmorphism.

Normative facial anthropometric and cephalometric growth curves, or ‘standards’, are central in assessing deviation from normal facial shape and treatment outcomes^1–4^. These are tabulated means and standard deviations of anthropometric measurements or mean cephalometric tracings, allowing clinical observations and measurements to be calibrated against what is expected for a given ancestry, age and sex. However, these approaches are limited. Cephalometric tracings are two-dimensional and cannot assess 3D shape. Traditional anthropometry only provides a limited assessment of facial shape as measurements between sparse anatomical landmarks. In contrast, the image processing technique of non-rigid 3D image registration can transfer many thousands of corresponding landmarks from a generic template image onto a 3D photograph of a subject rapidly and automatically^5,6^. In combination with geometric morphometric^7^ and dysmorphometric^8^ analysis, this enables average ‘expected’ faces or ‘archetypes’ of a given population, and statistical models of the variation therein^9–11^, to be constructed. Individual faces can be compared with this known variation^12–14^ to evaluate normal and abnormal facial shape. With a few exceptions (e.g.^15,16^), these have exclusively been applied in a research context, and by a limited number of research groups. The specialized tools, skills, and access to large normative datasets required to apply them has limited their use in clinic and research.

The 3D craniofacial growth curves developed here are sequences of age-specific expected faces and models of normal variation around the expected face. Conceptually, the expected face can be thought of as an average face. However, for reasons described in Supplementary Information 2, Section 2.1.3, we do not use the arithmetic average face and, therefore, use the more general term ‘expected’ face. These are constructed for each sex, based on a large normative database of 3D facial images. Alongside these, we provide a toolbox for assessing individuals against the models. In the remainder of this article we present the growth curves, and demonstrate their utility for assessment of individual patients.

## Methods

### Ethical approval

The study was approved by the ethical review board of KU Leuven and University Hospitals Gasthuisberg, Leuven (S56392) and data collection at various centers was approved by: Royal Children’s Hospital, Australia IRB (29008I); University of Pittsburgh IRB (PRO09060553 and RB0405013); Seattle Children’s Hospital IRB (12107); University of Texas Health Committee for the Protection Human Subjects (HSC-DB-09-0508); University of Iowa Human Subjects Office (200912764 and 200710721); Pennsylvania State University IRB (13103, 45727, 2503, 44929, 4320 and 1278); University of Cincinnati IRB (2015-3073); Indiana University–Purdue University Indianapolis IRB (1409306349); University College London Hospital IRB (JREC00/E042); and Sheffield Children’s Hospital IRB (MREC/03/4/022). All methods were carried out in accordance with the relevant guidelines and regulations.

### Subjects

#### Normative sample

**Figure 1:**
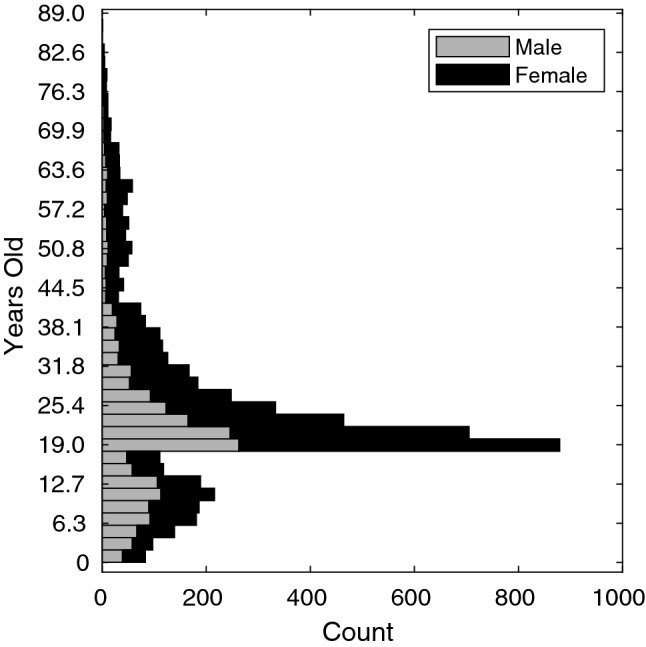
Demographics of the normative sample used in this study. This figure was generated in MATLAB 2021a (https://www.mathworks.com/).

The normative data used in this study are 5,443 images sourced from 4 collections from different centers: the Australian Head Examination and Assessment Database (AHEAD; N = 853) from the Royal Children’s Hospital, Melbourne, Australia; the online 3D Facial Norms database (3DFN; N = 1886; https://www.facebase.org/facial_norms/); collections from Indiana University–Purdue University Indianapolis (IUPUI; N = 730) and Pennsylvania State University (PSU; N = 1973). For the 3DFN, IUPUI and PSU databases, subjects of European genomic ancestry were selected as described in^17^. For the AHEAD subjects only self-reported ancestry was available. Only subjects of self-reported ‘white’ or ‘Australian’ ancestry or ancestry from any European country were included. These ancestry-based inclusion criteria were applied for this first iteration of the growth curves because of minimal data availability for other ancestries. The distributions of age and sex are shown in Fig. 1.

#### Patient samples

Patients included 9 male patients with Wolf Hirschhorn syndrome (WHS) (aged 0.4, 2.5, 5.2, 8.8, 9, 11.3, 14.1, 14.6, 18.1 years of age) collected at family support group meetings in the United Kingdom and the United States. All had a confirmed terminal deletion of 4p with a breakpoint more proximal than 4p16.3. These were drawn from a sample of WHS with ‘large’ deletions, analyzed previously, that were shown to have stronger facial phenotypes than those with smaller deletions^13^. Patients also included two with rare monogenic conditions imaged at the Department of Human Genetics, KU Leuven (1) a 6.7 years-old boy with a de novo* KAT6A* mutation c.4421_4422del (p.Cys1474Serfs*6) (NM_001099412.1) with clinically recognisable dysmorphism within the spectrum of Ohdo syndrome^18^ and (2) a 6.2 years-old girl with craniofrontonasal dysplasia (CFNS) caused by a de novo mutation within the *EFNB1*-gene: c.451 G>A (p.Gly 151 Ser) (NM_004429 4).

All subjects gave informed consent. If they were aged under 18 years or otherwise unable to give informed consent then consent was obtained on their behalf from their legal guardian. The two patients collected at Leuven consented for their images to be shown without facial coloring.

### Image quality control

Images were cleaned to remove extra-facial areas and any obscuring hair on the forehead. Any images with facial hair or a non-neutral facial expression were discarded. A facial expression was considered non-neutral if the individual was laughing, crying, smiling or otherwise emoting. The lips could be parted and the mouth partially open. Each image was manually annotated with five points placed approximately in the inner corners of the eyes, on the tip of the nose and at the corners of the mouth. These served to roughly initialize the non-rigid registration described in the following paragraph.

### Automatic dense quasi-landmark indication with non-rigid registration

So that multiple images can be combined and compared meaningfully, they are represented as a standard set of points (quasi-landmarks) that correspond anatomically across all images. To obtain this representation we use the open-source implementation ‘MeshMonk’ for automatic non-rigid image registration^5,6^ (https://github.com/TheWebMonks/meshmonk). This process deforms a generic template face, in this case comprising 7160 points, into the shape of each image. The deformed template, produced for each face, is the representation of each face as a standard set of points. All images in the normative and patient samples were processed in MeshMonk. The accuracy of the correspondence was assessed visually, and registration failures were discarded. The values reported reflect the numbers after unacceptable images and registration failures were removed.

The accuracy of this process was previously assessed on adult European faces without known genetic disorders or craniofacial dysmorphism^5^. In Supplementary Information 2, Section 1 we assessed the performance of MeshMonk on a sample including cases of a variety of genetic disorders with characteristic dysmorphism, as well as controls. We assessed the accuracy of the correspondence at specific landmark points. Median error ranged from 1.08 mm for the pronasale to 3.98 mm for the gonion. The registration failed on 5.5% of the 289 images.

### Building ‘3D growth curves’

The growth curves were trained from 5443 males and females aged between 0.05 and 88 years from the normative sample described above. Full computational details are given in Supplementary Information 2, Section 2, and the code is made available at https://github.com/harrymatthews50/3DGrowthCurves. Briefly, the 3D growth curves comprise a series of age and sex specific expected facial shapes and parameterizations of the variation around the expected shape. Facial shape was represented as a n (observations) x k variables matrix, where the k variables are x,y and z co-ordinates of the 7160 quasi-landmarks imposed on each face, after generalized Procrustes analysis was performed to remove non-shape related variation. To estimate the expected facial shape, we used locally weighted (kernel) linear regression^19,20^. The local regression model was a partial-least-squares (PLS) regression of facial shape onto age^21^ and the weighting function was Gaussian (see Supplementary Information 2, Sections 2.1.2 and 2.1.3). The weighting function had a variable width (standard deviation of the Gaussian) so as to accommodate the varying amounts of training data available at different ages while ensuring a representative model of normal variation (see Supplementary Information 2, Section 2.2). Essentially this models facial shape as a non-parametric, non-linear function of age as was done in Matthews et al.^22^.

We estimate normal facial variation for each age and sex within the residuals of a similarly weighted PLS-regression model with training faces recentered on the expected face for that age and sex (Supplementary Information 2, Section 2.1.4). The residuals of this regression model constitute age-adjusted displacements of each point on each face from the expected face, weighted according to the Gaussian weighting function. Variation at each point on the face was calculated as the root-mean-square (RMS) of the displacements at each point in the anterior–posterior; superior-inferior; and medial–lateral anatomical directions separately. RMS was also calculated along the direction normal to the surface of the expected face at each point, as well as the total magnitude of the displacement. RMS values were then scaled by the inverse of the sum of the weights. Hereafter we refer to these as ‘pointwise standard deviations’. See Supplementary Information 2, Section 2.1.5.1 for further details.

Variation at each age and sex was also modelled as separate statistical shape models (SSMs). A SSM comprises modes of variation (which are linear transformations of facial shape) and the expected variation along each mode. Modes of variation theoretically span all plausible realistic faces for a given age and sex and can be used to determine faces or facial regions that fall outside of this span. Each SSM was fitted using a singular value decomposition of the residuals described in the previous paragraph (Supplementary Information 2, Section 2.1.5.2), without column centering or standardization. For each SSM we retained the components explaining 98% of the variation.

As the normative training data cannot be made fully available, at https://github.com/harrymatthews50/3DGrowthCurves we provide models (SSMs and pointwise standard deviations) evaluated for every 0.3 years of age between 0.5 and 68.9 years for females and 0.5–51.8 years for males (see Supplementary Information 2, Section 2.2 for the rationale for the differing age ranges).

### Individual patient assessments

To demonstrate the utility of the growth curves for the assessment of individual patients, we assess three average patient ‘morphs’ intended to represent the typical facies of a given disorder (Wolf Hirschhorn syndrome; WHS) at different ages. We also assess similarly constructed morphs of unaffected individuals. ‘Morphs’ here refers to average faces of a small number of individuals, constructed by averaging corresponding co-ordinates of the faces after co-alignment by generalized Procrustes analysis (GPA). Faces were scaled to unit size during the GPA to average the shapes of the images, independently of size. They were then scaled back to a realistic size (the average size of the faces comprising each average). WHS morphs were each composed of three male patients with WHS (ages = (a) 2.5, 5.2, 0.4; (b) 11.3, 9, 8.8; and (c) 18.1, 14.6, 14.1 years of age). Each of the three unaffected morphs was composed of three individuals randomly drawn from the normative sample and constrained to be aged within ± 0.5 years of age 2, 10 and 20 respectively. We also assess the two individual patients with rare monogenic conditions. Using the growth curves, the phenotype of an individual can be visualized and numerically described as its facial signature. This indicates how far each point on the face is from the corresponding point on their age- and sex-specific expected face and is represented as a z-score (computed based on the pointwise standard deviation)^12^. We produce facial signatures for each morph and individual patient against their age and sex-specific model of variation. The age of a morph was designated as the mean age of the faces comprising it.

The age and sex-specific SSMs can be used to also create ‘normal equivalents’^14^. To produce a normal equivalent of a patient, regions of the face that do not fit within the span of normal faces are identified and interpolated based on the regions that do fit. Specifically, the patient is projected onto the modes of variation of their age- and sex-specific SSM and reconstructed from these projections. The projection is constrained so as to not exceed a critical Mahalanobis distance to the origin, corresponding to a p-value of 0.05 (Supplementary Information 2, Section 3.1.2.3). The projection is also weighted (Supplementary Information 2, Section 4.3), and these weights are updated over successive iterations so that the regions of the face that were not well reconstructed on the previous iteration are given less weight in the next iteration. One iteration of this algorithm with all equal weights is equivalent to the ‘closest control’ algorithm^23^. The result is a representation of the patient with the abnormal component of their face removed and replaced with the most statistically plausible equivalent. Whereas the facial signatures are intended to assess subtler deviations from the expected shape and are more suited to identifying facial characteristics that may or may not lie within normal range variation; the normal equivalent was developed primarily to produce a synthetic correction of extreme and localized abnormalities, such as in patients undergoing orthognathic treatments, to assist with treatment planning and assessment. To demonstrate the principle, we produce a normal equivalent of the youngest male patient morph using the SSM for 40 year-old males. Within the population of 40 year-old male faces this juvenile male face is expected to be extreme and abnormal in some aspects.

The 9 individuals from the normative sample used to create the unaffected morphs were excluded from the data used to train the growth curves used for these assessments, although they are included in the training data of the growth curves distributed with this paper. Full computational details are given in Supplementary Information 2, Section 3. Code is made available at the GitHub link above, including a MATLAB script whereby new patients can be assessed against the models of normal variation.

## Results

### 3D growth curves

**Figure 2:**
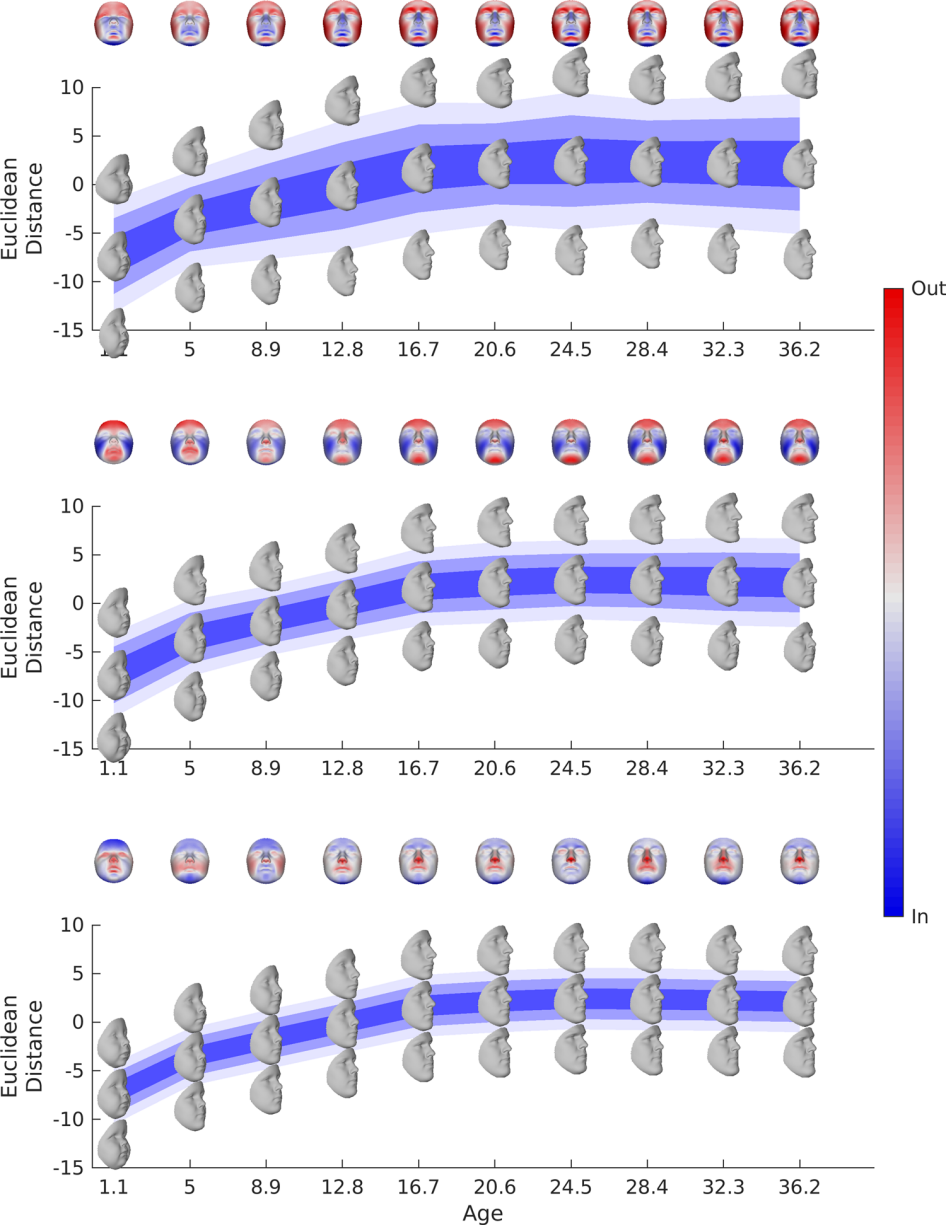
Age-specific statistical shape models for males. This figure shows the first 3 modes of variation (top to bottom) for males at some example ages (horizontal axis). The faces in the center of each panel are the expected faces and their position along the vertical axis is their projection on the first mode of variation of the expected faces. Change in the position of these faces on this axis represents the change in shape of the expected faces along this aspect of variation in Euclidean distance (square root of the sum of squared differences between all pairs of corresponding points). The outermost faces are the expected faces morphed to plus or minus three standard deviations along the mode of variation (mode 1-mode 3; top to bottom). The graded colors represent the span of  ± 1(dark) ±  2 (lighter) and 3 (lightest) standard deviations, also in Euclidean distance. This figure was generated in MATLAB 2021a (https://www.mathworks.com/).

**Figure 3:**
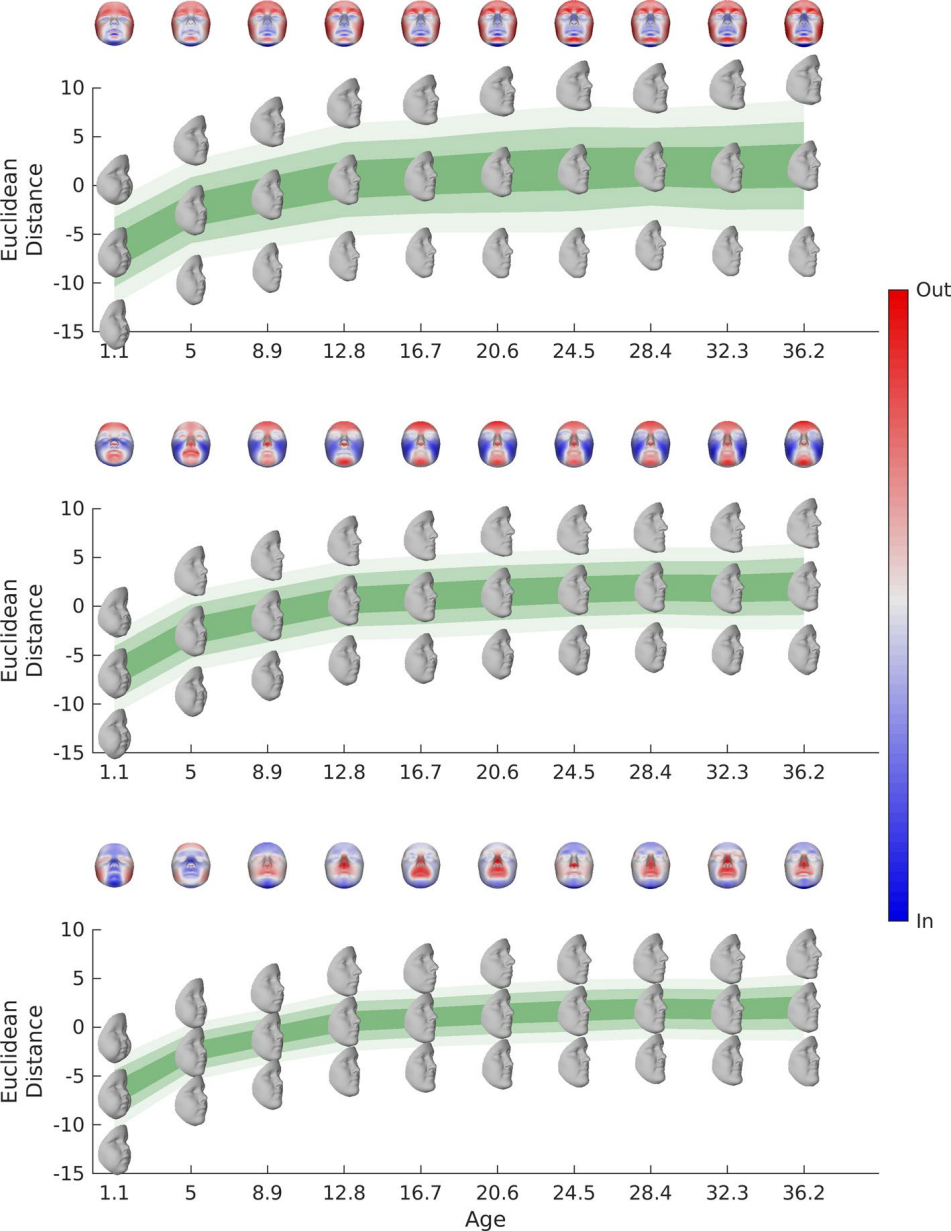
Age-specific statistical shape models for females. This figure shows the first 3 modes of variation (top to bottom) for females at some example ages (horizontal axis). The faces in the center of each panel are the expected faces and their position along the vertical axis is their projection on the first mode of variation of the expected faces. Change in the position of these faces on this axis represents the change in shape of the expected faces along this aspect of variation in Euclidean distance (square root of the sum of squared differences between all pairs of corresponding points). The outermost faces are the expected faces morphed to plus or minus three standard deviations along the mode of variation (mode 1–mode 3; top to bottom). The graded colors represent the span of ± 1(dark) ± 2 (lighter) and 3 (lightest) standard deviations, also in Euclidean distance. This figure was generated in MATLAB 2021a (https://www.mathworks.com/).

Figures 2 and 3 show the expected faces and first three modes of variation (top to bottom) for males and females respectively at some example ages. The notable changes in the expected faces, spread, and (more subtly) changes in the aspects of variation modeled by the modes at different ages and between the sexes highlight important differences in the underlying covariance structure and emphasizes the need to make age-specific models.

**Figure 4:**
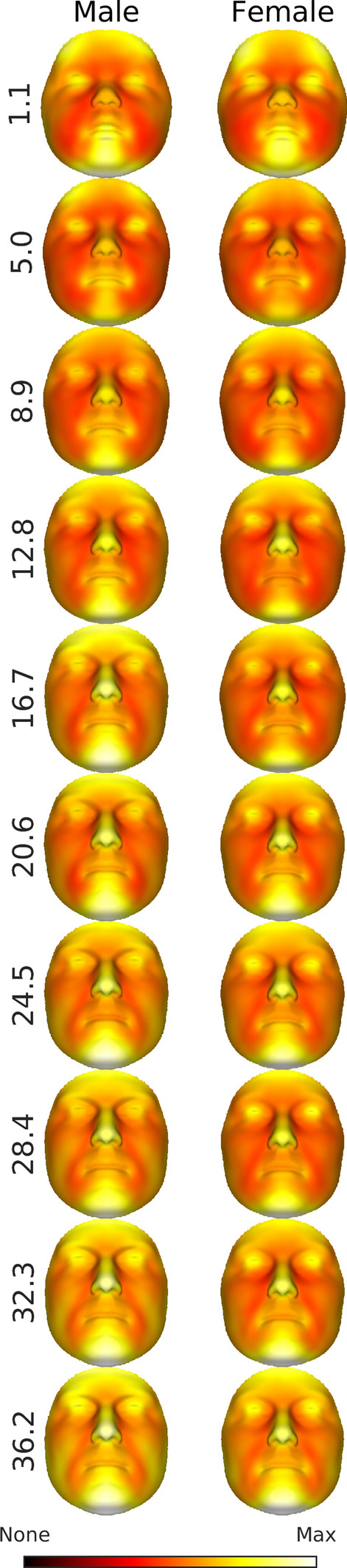
Root mean-square variation around the expected faces for some example ages. All color-maps use the same color-scale and are therefore comparable visually. This figure was generated in MATLAB 2021a (https://www.mathworks.com/).

Each mode of variation only represents a particular aspect of the variation structure. The change in the overall variations for males and females is illustrated in Fig. 4. In this plot the expected faces are colored according to the root-mean-squared 3D displacement of each point from the expected face. The strongest variation is in the shape of the chin, nose, eyes and forehead. In general, the amount of variation increases with age and, at most ages, is greater for males than for females.

### Illustrative patient assessments

**Figure 5:**
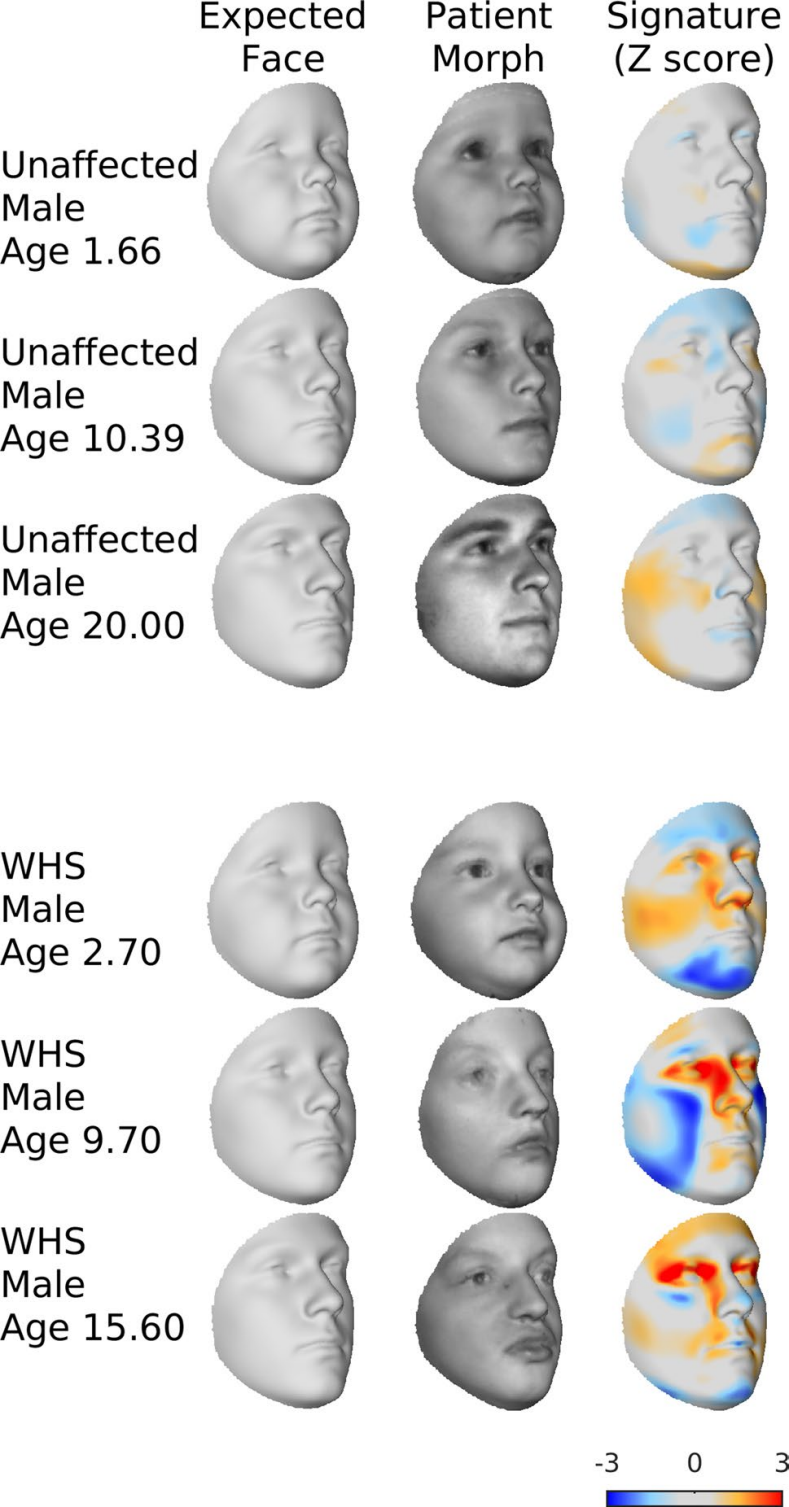
Assessing average patient morphs against the 3D growth curves. The first column plots the age and sex-specific expected face for the age and sex of the patient. The second plots the 3D image of the patient morph. The third plots the facial signature of the patient morph along the direction perpendicular to the expected face. Each point is colored according to the patient morph’s individual z-score. Orange and red indicates the region of the face is displaced outwardly, relative to the expected facial shape, light and dark blue indicate the point is displaced inwardly. Dark red and dark blue indicate z scores outside of the range ± 2. This figure was generated in MATLAB 2021a (https://www.mathworks.com/).

Figure 5 illustrates the assessments of the patient morphs. As these patient morphs are average faces (of 3 participants) they are expected to deviate less from the expected faces than will individual patients. WHS is characterized by a prominent glabella, continuous with the forehead, arched eyebrows, hypertelorism and broad prominent nose. This gestalt is sometimes hard to recognize at birth, especially in cases with only small deletions, and in infants and becomes more obvious with age. This is consistent with the assessments of the WHS morphs. The signature of the youngest morph shows milder (relative to the older morphs) hypertelorism, and nasal widening as shown by the red on the inner and outer corners of the eye and the lateral sidewalls of the nose. In the two older morphs, the signatures on these regions are darker red as the characteristic features are more pronounced. In contrast, and as expected, these characteristic features are not seen in the assessment of the unaffected morphs. The value of using age-specific models for this assessment is demonstrated by contrasting this figure to Supplementary Information 1. Here instead of using an age and sex-specific model, each patient morph was assessed against a model constructed from the whole training data, with each training observation given equal weight. For the youngest (WHS and unaffected) morphs, characteristically juvenile features including pronounced buccal fat and chin retrognathia (relative to expected adult morphology) are highlighted obscuring the characteristic WHS features of the WHS morph.

**Figure 6:**
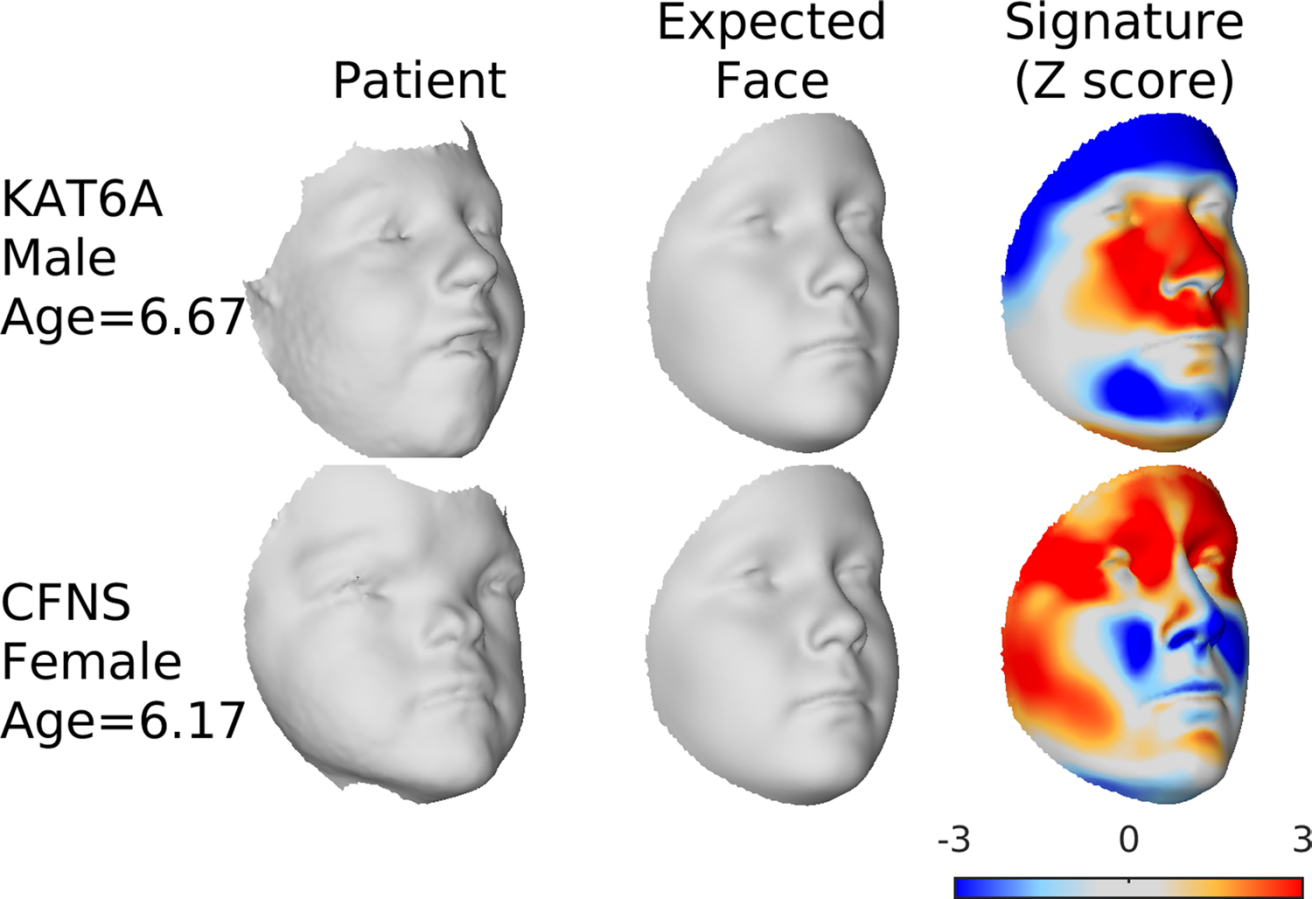
Assessing individual patients. The first column plots the age and sex-specific expected face for the age and sex of the patient. The second plots the 3D image of the patient morph. The third plots the facial signature of the patient morph along the direction perpendicular to the expected face. Each point is colored according to the patient morph’s individual z-score. Orange and red indicates the region of the face is displaced outwardly, relative to the expected facial shape, light and dark blue indicate the point is displaced inwardly. Dark red and dark blue indicate z scores outside of the range  ± 2. This figure was generated in MATLAB 2021a (https://www.mathworks.com/).

Figure 6 shows the assessment of two individual patients. The first has a de novo* KAT6A* mutation and presented with clinically recognisable dysmorphism within the spectrum of Ohdo syndrome^18^. Typical features caused by *KAT6A* mutations are visible in the signature: a broad nasal tip and a prominent nasal bridge (outward displacement of the nose and area around the nose) and bi-temporal narrowing (inward displacement of the bi-temporal region). The second patient has craniofrontonasal dysplasia (CFNS) caused by a de novo* EFNB1* mutation^24^. Also, for this patient, the facial signature clearly shows typical dysmorphic features: i.e. hypertelorism and a wide face at the level of the lateral zygomata (outward displacement of the peri-orbital region), short nose (inward displacement of the nasal tip) and maxillary hypoplasia (inward displacement at the level of the maxillae).

**Figure 7:**
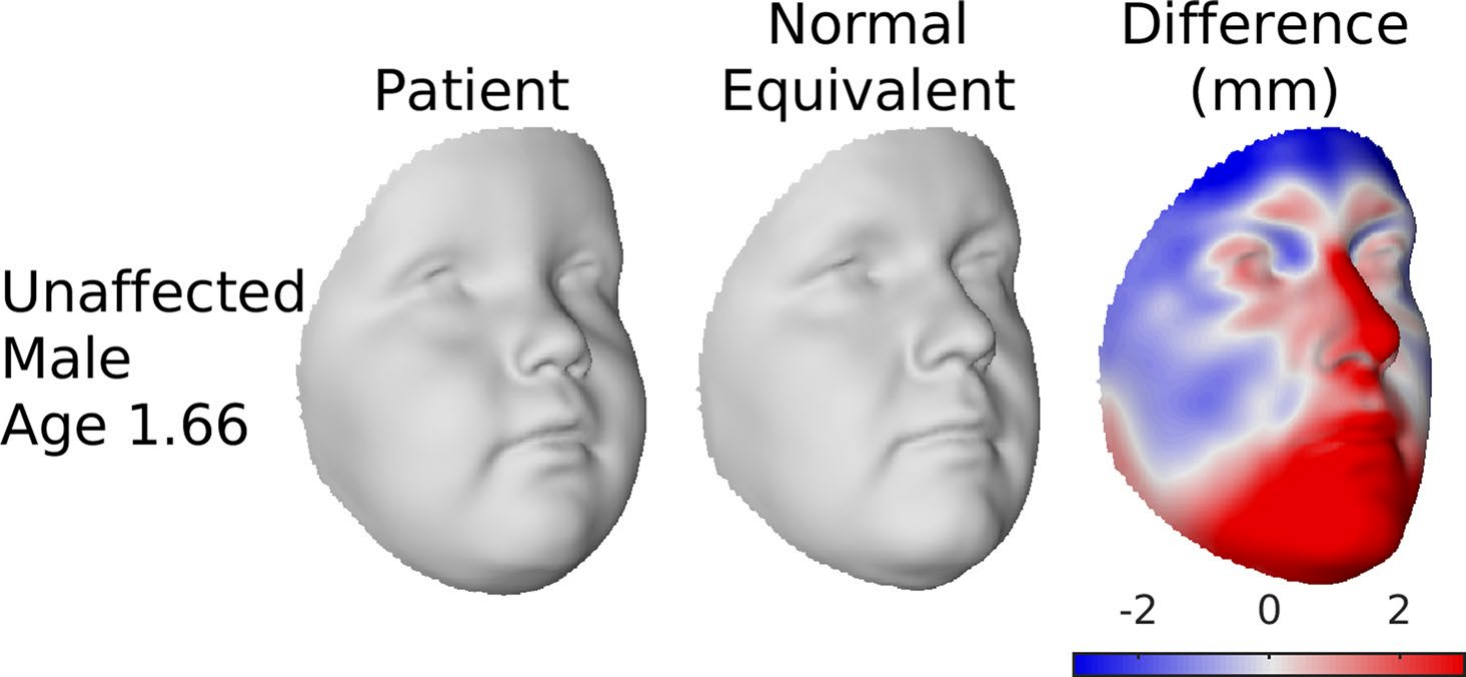
Normal equivalent of unaffected 1.66 year old male average patient morph. The first column shows the patient. The second is their normal equivalent derived using an SSM of 40 year old males. The third shows the difference between the patient and the normal equivalent in mm in the direction perpendicular to the facial surface. The effect is that the typically juvenile features of the morph (a more retrognathic chin and a less prominent nose) are replaced with the more adult features in the normal equivalent. This figure was generated in MATLAB 2021a (https://www.mathworks.com/).

Figure 7 demonstrates the normal equivalent algorithm. In the normal equivalent of the 1.66 years-old unaffected male morph, derived using the model for 40 year-old males, the characteristically juvenile features (chin retrognathia and pronounced buccal fat) of the morph are interpolated across with more adult features. This is because, in the context of the model, which codes for only adult facial variation, these features are outside of the span of normal variation.

## Discussion

As more institutions collect 3D images as part of routine clinical care, there is a need for methods of facial assessment that take full advantage of the information available in such images. In this article, we derive and present ‘3D growth curves’ of craniofacial shape to augment and inform visual and landmark-based assessment of individual patients and cohorts. We provide these along with a toolbox for assessing patients against these models and demonstrate their utility for this purpose.

Previous work also used local kernel-based regression methods to explore non-linear changes in facial shape as a function of age^22,25^ and to model changes in variation structure in non-shape data over time^26^. Methodologically, this paper synthesizes these techniques to model jointly the change in typical facial shape and variation with age. These open-source models of changing facial shape and variation are unique and can facilitate the assessment of facial abnormality by the broader research and clinical community. The remainder of the discussion considers this article in the context of these intended applications.

To assess facial characteristics of an individual observations of, or measurements derived from, a patient need to be calibrated against what is normal and expected for a given age. For example, the mandible and chin of almost all infants is retro-positioned relative to the upper-lip and maxilla, so the assessment of retrognathia for infants must be calibrated against this known variability. In general, objective approaches can be expected to be both more accurate and reliable than visual assessment. Univariate growth curves of inter-landmark measurements, such as those developed by Leslie Farkas^1^, address this issue to a degree. These outsource the cognitively complex task of calibration against age and sex appropriate normal variation to comparison with numerical normative reference values. However, the collection of these measurements on large normative datasets, often by multiple operators, can still be subject to substantial inter- and intra-operator error, are time-consuming to collect and are limited to the assessment of aspects of craniofacial shape that are easily described by such measurements. Further, given the development of different facial anatomical structures is interconnected, a single feature rarely changes in isolation. Therefore holistic assessment of the full face is preferable for describing, quantifying and analyzing disturbances of craniofacial development. To achieve this, we employ non-rigid surface registration to automatically indicate thousands of landmarks onto each image used to train the growth curves, and all images to be assessed. This approach shows sub-millimeter repeat-measurement variation^27^ and captures the entirety of facial shape, not just those aspects described by inter-landmark distances. Through the use of these growth curves and the automatic registration strategy, clinicians and researchers will be able to perform holistic assessments that are standardized and repeatable across time and operators.

Analogous to univariate growth curves, the 3D growth curves comprise, for each sex, a series of age-appropriate typical facial shape and variation around it. These parameterize normal variation in two ways. The first is as the point-by-point standard deviation of 3D displacements from the expected face. The second is as a statistical shape model (SSM), which models the span of statistically plausible faces at a given age. Although many open-source statistical shape models of the human face exist^11^, these are devised in the context of computer vision applications and are made of combined samples of children and adults, often include expression variation, and are not integrated with tools for medical assessment. The core strength of our growth curves over these existing models is that they are constructed to be age-specific and come integrated with a toolbox for performing patient assessments.

The growth curves and toolbox allow clinicians and researchers to perform state-of-the art assessments of facial abnormality without collecting large databases of normal-range faces in-house. The pointwise standard deviations allow each point on a new face to be described as a z-score, defining a color-coded ‘map’, that highlights the points that deviate from typical facial shape. These ‘facial signatures’ have proven utility in research to characterize phenotypes of individuals, and variation among phenotypes^12,13^. The SSM can be used to synthesize a ‘normal equivalent’ of the subject which is an approximation of the subject that lies within the span of normal facial variation^8,14,15^. This can be used as a guide to surgical interventions to correct craniofacial abnormalities. Using morphs and individual patients we showed that the assessments using the growth curves can isolate characteristic features of disorders independent of variations due to normal facial growth, thereby satisfying the essential purpose of a growth curve.

At present these assessments can be performed with a custom-built toolbox implemented in the proprietary MATLAB programming language. With input from collaborators who have minimal computer science or engineering experience, clinicians should be able to incorporate them into clinical assessments and research programs. In the future, they can be incorporated into more user-friendly open-source graphical user interfaces^28^, removing the need for any programming expertise on the part of the user.

Bio-informatics tools are already complementing clinical diagnosis and, with continual advances in deep-learning, this trend is likely to continue. One of the forerunners in this field is the Face2Gene app developed by FDNA. Face2Gene employs the ‘deep gestalt’ framework^29^ to extract features from a 2D image of a patient and compare them to pre-learned 2D facial ‘gestalts’ composed from 2D images of individuals with known genetic disorders. Based on similarity to these gestalts, it prioritizes candidate disorders for the individual. In contrast, the 3D growth curves presented here are intended to facilitate phenotypic analysis and description by generating a comprehensive numerical and visual description of the phenotype of an individual patient. This can facilitate description of atypical cases of a given disorder and those with extremely rare (for which sufficient training images are not available for Face2Gene to learn the facial gestalts) or yet unknown disorders. In future, with growing numbers of 3D images of patients with genetic disorders being collected (e.g. https://www.facebase.org/chaise/record/#1/isa:dataset/RID=TJ0), analogous approaches to automatic diagnosis based on 3D images (e.g. Hallgrimsson et al.^30^) can be expected to outperform those based on 2D images. Although not aimed at automated diagnosis per se, the work of this study can be incorporated into this future work by extracting confounding variation due to age and sex from training images and the images being assessed.

An important limitation in our study is the lack of ethnic diversity in the growth curves. We included only cases of European ancestry based on genomic ancestry, or self-reported ‘European’, ‘Australian’ or ‘White’ ancestry. Facial shape and variation will vary depending on ancestry and, at present, there is a paucity of data to correctly model variations within different ancestries as well as across different ages. With growing databases of facial images and paired genomic data, an important next step will be to define models that can be evaluated not only for age and sex but for a given genomic ancestry. In this paper, we define a general framework for building these growth curves as such data become available.

This paper derived and presented ‘3D growth curves’ of human facial shape. These are integrated with a toolbox that enables patients to be assessed for facial abnormality with respect to these growth curves. By making these openly available, we aim to enable clinicians and researchers to perform standardized and objective facial assessments without requiring large in-house databases of normal-range faces or in-house software.

## Supplementary Information

Supplementary Information 1.

Supplementary Information 2.

## Data Availability

Growth curves derived from the normative databases are available at https://github.com/harrymatthews50/3DGrowthCurves along with the patient morphs and all custom code necessary for performing facial assessments using the growth curves. Other underlying code is available as part of MATLAB software, available through MathWorks (https://www.mathworks.com). ~ 35% of the normative sample (the faces from the 3DFN database) are available from the FaceBase repository pending an approved data usage agreement (https://www.facebase.org/facial_norms/). The other normative databases and individual patient images were collected without consent for broad data sharing and are not publicly available. However access to these is not needed to fully explore and deploy the results of this work.
