# Supplementary material for: Large-scale open-source three-dimensional growth curves for clinical facial assessment and objective description of facial dysmorphism
Source: Sci Rep. 2021 Jun 9;11:12175. doi: 10.1038/s41598-021-91465-z (PMC8190313; doi:10.1038/s41598-021-91465-z)
Supplement: Supplementary file 1 — Supplementary Information 1. [file 41598_2021_91465_MOESM1_ESM.pdf]

Large-scale open-source three-dimensional growth curves for clinical facial assessment and objective description of facial dysmorphism

Short title: 3D growth curves for facial assessment

Harold S Matthews[1,2,3,*] PhD, Richard L Palmer[4] PhD, Gareth S Baynam[4,5,6,7] MD, PHD, Oliver W Quarrell[8] MD, Ophir D Klein[9] MD, PhD, Richard A Spritz[10], MD, Raoul C Hennekam[11] MD PhD, Susan Walsh[12] PhD, Mark Shriver PhD[13], Seth M Weinberg[14] PhD, Benedikt Hallgrimsson[15] PhD, Peter Hammond[1] PhD, Anthony J Penington[3,16,17] MD(res), Hilde Peeters[1] MD PhD, Peter D Claes[1,2,3,18] PhD

1 Department of Human Genetics, KU Leuven, 3000 Leuven, Belgium; 2 Medical Imaging Research Center, UZ Leuven, 3000 Leuven, Belgium; 3 Facial Sciences Research Group, Murdoch Children's Research Institute, Parkville, 3052, Australia; 4 School of Earth and Planetary Sciences, Faculty of Science and Engineering, Curtin University, Perth, 6845, Australia; 5 Western Australian Register of Developmental Anomalies, King Edward Memorial Hospital, Perth, Australia; 6 Telethon Kids Institute and Division of Paediatrics, Faculty of Health and Medical Sciences, University of Western Australia, Perth, Australia; 7 Faculty of Medicine, Notre Dame University, Fremantle, Australia; 8 Dept Clinical Genetics Sheffield Children's NHS Trust. OPDII Northern General Hospital, Herries Road, Sheffield S5 7AU, UK; 9 Program in Craniofacial Biology, Departments of Orofacial Sciences and Pediatrics, and Institute for Human Genetics, University of California, San Francisco, San Francisco, CA, USA 10 Human Medical Genetics and Genomics Program, University of Colorado School of Medicine, Aurora, CO USA; 11 Department of Pediatrics, Amsterdam University Medical Center, University of Amsterdam, Amsterdam, The Netherlands; 12 Department of Biology, Indiana University Purdue University Indianapolis, Indianapolis, IN 46202, USA; 13 Department of Anthropology, Pennsylvania State University, State College, PA, 16802, USA; 14 Center for Craniofacial and Dental Genetics, University of Pittsburgh, Pittsburgh, PA 15219, USA; 15 Department of Cell Biology & Anatomy, Cumming School of Medicine; Alberta Children's Hospital Research Institute, University of Calgary, Calgary, AB T2T 4N1; 16 Department of Plastic and Maxillofacial Surgery, Royal Children's Hospital, Melbourne, 3052 Australia; 17 Department of Pediatrics, University of Melbourne, Melbourne, 3052, Australia; 18 Department of Electrical Engineering, ESAT/PSI, KU Leuven, Leuven, 3000, Belgium

*Corresponding author
Medical Imaging Research Center
UZ Gasthuisberg
Herestraat 49
3000 Leuven
E-mail: harry.matthews@kuleuven.be

S1 Figure

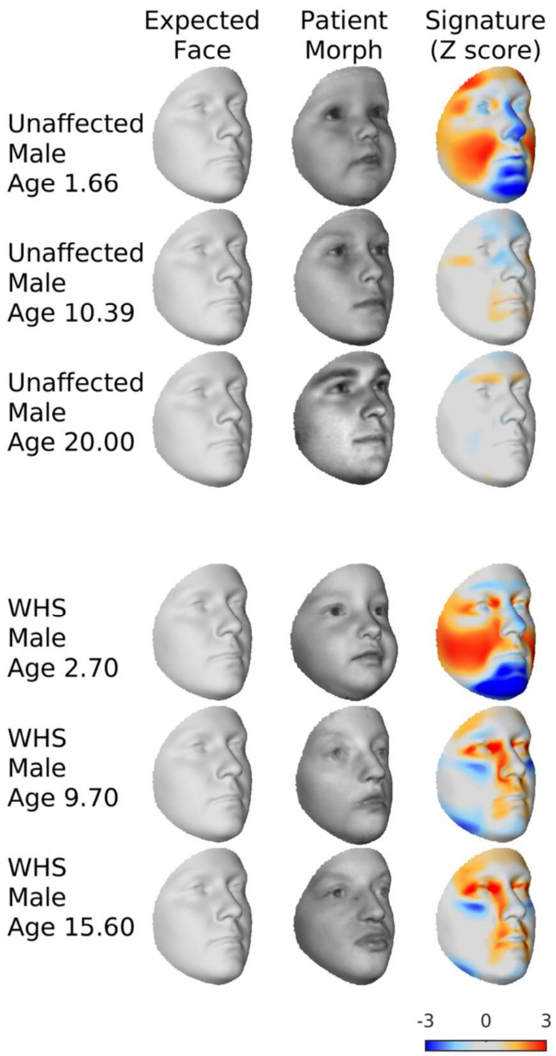

S1 Figure. Assessing individual patients against a model of variation derived from the entire normative training sample with equal weight given to each training observation. The first column plots the age and sex-specific expected face for the age and sex of the patient. The second plots the 3D image of the patient morph. The third plots the facial signature of the patient morph along the direction perpendicular to the expected face. Each point is colored according to the patient morph's individual z-score. Orange and red indicates the region of the face is displaced outwardly, relative to the expected facial shape, light and dark blue indicate the point is displaced inwardly. Dark red and dark blue indicate z scores outside of the range +/- 2. This figure was generated in MATLAB 2021a (https://www.mathworks.com/).
