# Supplementary material for: Large-scale open-source three-dimensional growth curves for clinical facial assessment and objective description of facial dysmorphism
Source: Sci Rep. 2021 Jun 9;11:12175. doi: 10.1038/s41598-021-91465-z (PMC8190313; doi:10.1038/s41598-021-91465-z)
Supplement: Supplementary file 2 — Supplementary Information 2. [file 41598_2021_91465_MOESM2_ESM.pdf]

Large-scale open-source three-dimensional growth curves for clinical facial assessment and objective description of facial dysmorphism

Short title: 3D growth curves for facial assessment

Harold S Matthews[1,2,3,*] PhD, Richard L Palmer[4] PhD, Gareth S Baynam[4,5,6,7] MD, PHD, Oliver W Quarrell[8] MD, Ophir D Klein[9] MD, PhD, Richard A Spritz[10], MD, Raoul C Hennekam[11] MD PhD, Susan Walsh[12] PhD, Mark Shriver PhD[13], Seth M Weinberg[14] PhD, Benedikt Hallgrimsson[15] PhD, Peter Hammond[1] PhD, Anthony J Penington[3,16,17] MD(res), Hilde Peeters[1] MD PhD, Peter D Claes[1,2,3,18] PhD

1 Department of Human Genetics, KU Leuven, 3000 Leuven, Belgium; 2 Medical Imaging Research Center, UZ Leuven, 3000 Leuven, Belgium; 3 Facial Sciences Research Group, Murdoch Children's Research Institute, Parkville, 3052, Australia; 4 School of Earth and Planetary Sciences, Faculty of Science and Engineering, Curtin University, Perth, 6845, Australia; 5 Western Australian Register of Developmental Anomalies, King Edward Memorial Hospital, Perth, Australia; 6 Telethon Kids Institute and Division of Paediatrics, Faculty of Health and Medical Sciences, University of Western Australia, Perth, Australia; 7 Faculty of Medicine, Notre Dame University, Fremantle, Australia; 8 Dept Clinical Genetics Sheffield Children's NHS Trust. OPDII Northern General Hospital, Herries Road, Sheffield S5 7AU, UK; 9 Program in Craniofacial Biology, Departments of Orofacial Sciences and Pediatrics, and Institute for Human Genetics, University of California, San Francisco, San Francisco, CA, USA 10 Human Medical Genetics and Genomics Program, University of Colorado School of Medicine, Aurora, CO USA; 11 Department of Pediatrics, Amsterdam University Medical Center, University of Amsterdam, Amsterdam, The Netherlands; 12 Department of Biology, Indiana University Purdue University Indianapolis, Indianapolis, IN 46202, USA; 13 Department of Anthropology, Pennsylvania State University, State College, PA, 16802, USA; 14 Center for Craniofacial and Dental Genetics, University of Pittsburgh, Pittsburgh, PA 15219, USA; 15 Department of Cell Biology & Anatomy, Cumming School of Medicine; Alberta Children's Hospital Research Institute, University of Calgary, Calgary, AB T2T 4N1; 16 Department of Plastic and Maxillofacial Surgery, Royal Children's Hospital, Melbourne, 3052 Australia; 17 Department of Pediatrics, University of Melbourne, Melbourne, 3052, Australia; 18 Department of Electrical Engineering, ESAT/PSI, KU Leuven, Leuven, 3000, Belgium

*Corresponding author
Medical Imaging Research Center
UZ Gasthuisberg
Herestraat 49
3000 Leuven
E-mail: harry.matthews@kuleuven.be

S1 Text

Table of Contents

1 Accuracy and robustness of 'MeshMonk' surface registration on dysmorphic faces 3

1.1 Background and Rationale 3

1.2 Learning anatomical loci on the template mesh 4

1.3 Assessing registration failures and precision of the achieved correspondences 6

1.3.1 Test sample 6

1.3.2 Image processing and non-rigid registration 7

1.3.3 Visual assessment of registration failures 7

1.3.4 Types of registration failures 8

1.3.5 Frequencies of registration failures 9

1.3.6 Anatomical precision of the mapping 10

2 Constructing age appropriate models of normal variation 13

2.1 Constructing models of normal variation. 13

2.1.1 General 13

2.1.2 Computing observation weights 13

2.1.3 Computing the expected face 14

2.1.4 Weighting, re-centering and statistical age adjustment 15

2.1.5 Modelling normal variation 15

2.2 Age-adaptive bandwidth setting 17

3 Patient Assessment from models of normal facial variation 19

3.1 Patient assessment using age-appropriate models 19

3.1.1 Facial Signatures 19

3.1.2 Normal equivalent 20

4 Further technical details 22

4.1 Calculating outlier weights 22

4.2 Weighted Procrustes transformation 23

4.3 Weighted projection onto the statistical shape model. 24

4.4 Computing reconstruction error 26

5 References 27

1 Accuracy and robustness of 'MeshMonk' surface registration on dysmorphic faces

1.1 Background and Rationale

The purpose of spatially-dense non-rigid image registration is to automatically impose a standard number of points onto each image such that the anatomical meaning of each point in one image is the same as in all the others. E.g. if point 400 is on the tip of the nose in one image it should be on the tip of the nose in all images. This is accomplished by gradually warping a standard template face into the shape of each face to be analyzed. This is done is such a way that the points of the template retain their anatomical meaning during the warping so that (e.g.) the tip of the nose of the template ends up on the tip of the nose on the target. The co-ordinates of these points now represent the shape of the target face using a standard set of points. When this is applied to many faces it allows for their shapes to be compared and combined for multivariate analysis.

In this work we apply the open source MeshMonk toolbox for performing this non-rigid registration. This toolbox was initially tested (1) using adult European faces. In this supplement we assess its performance on faces with various facial dysmorphisms, of varying ages including children. To do so we: 1. learn which points of the template correspond to particular anatomical loci and 2. visually assess the resulting positions on a sample of target faces to identify gross failures of the registration algorithm and report their success rate. We then compare these resulting positions to landmark points that have been manually annotated on the images. These manual annotations are considered a quasi 'ground truth'

for anatomical correspondence, which are compared to the correspondence induced by the registration.

1.2 Learning anatomical loci on the template mesh

For visualization and validation of the resulting correspondences it is necessary to know which positions of the template mesh correspond to particular anatomical loci. For this, 10 faces without known genetic disorders or craniofacial trauma, not otherwise used in the study, were manually annotated with 20 landmarks by author PH. The demographic characteristics of these individuals is shown in S1 Text Table 1. These faces were non-rigidly registered with the MeshMonk pipeline, producing a version of the face that shares the template topology, but is in the shape of target face. The locations of the landmarks on each target face were recorded as their barycentric co-ordinates in terms of the three closest points on the registered version of the face. These 10 landmark indications were then transferred into the 3D Cartesian co-ordinate system of the original unwarped template mesh, by reconstructing their Cartesian co-ordinates from the barycentric co-ordinates. These 10 landmark indications were averaged and projected to the surface of the template mesh. These averaged locations were then again recorded as barycentric co-ordinates, giving a robust indication of the positions of these landmarks in terms of the template topology. The locations of these landmarks on the template are shown in S1 Text Figure 1. Once these locations are known and coded in this way on the template they can be easily visualized on any registered face and compared to manual landmark placements to assess the accuracy of the correspondences visually and quantitatively.

S1 Text Table 1 Demographics of 10 individuals used to learn the anatomical locations of the landmarks.

Subject	Age (years)	Sex
1	58.2	M
2	73.5	M
3	30.6	M
4	44.4	M
5	39.2	M
6	51.1	F
7	38.5	M
8	39.8	F
9	44.7	M
10	32	M

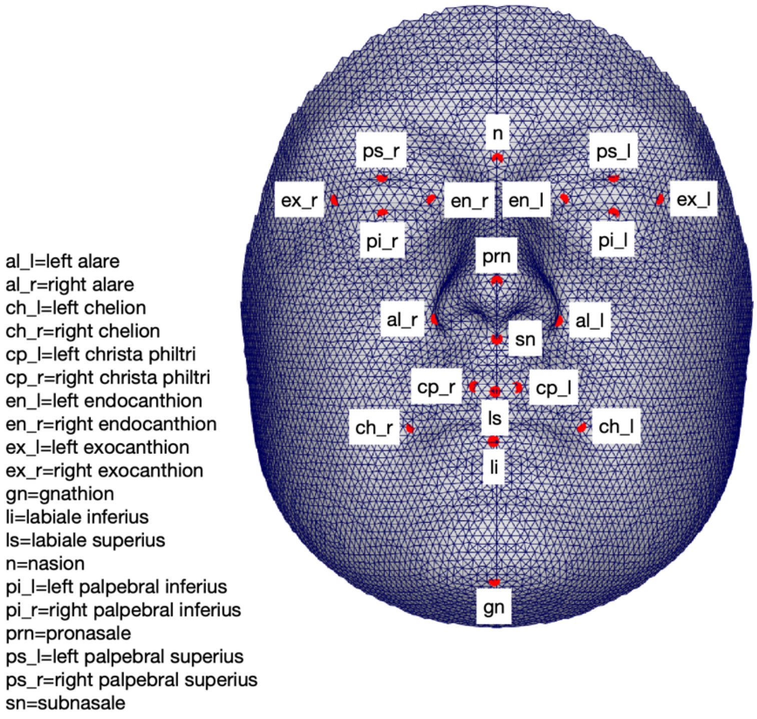

S1 Text Figure 1 Locations on template mesh, corresponding to anatomical landmarks. This figure was generated in MATLAB 2021a (https://www.mathworks.com/).

1.3 Assessing registration failures and precision of the achieved correspondences

1.3.1 Test sample

The test sample comprised individuals with various disorders affecting craniofacial shape as well as a control sample without known genetic conditions. Each image was visually screened and faces with facial hair or a non-neutral facial expression were excluded. A facial expression was considered 'neutral' if the individual was not laughing, crying, smiling or otherwise emoting. The lips were sometimes parted and the mouth open. S1 Text Figure 2 displays demographic characteristics of this sample. Each face was manually annotated with the same 20 landmarks by author PH.

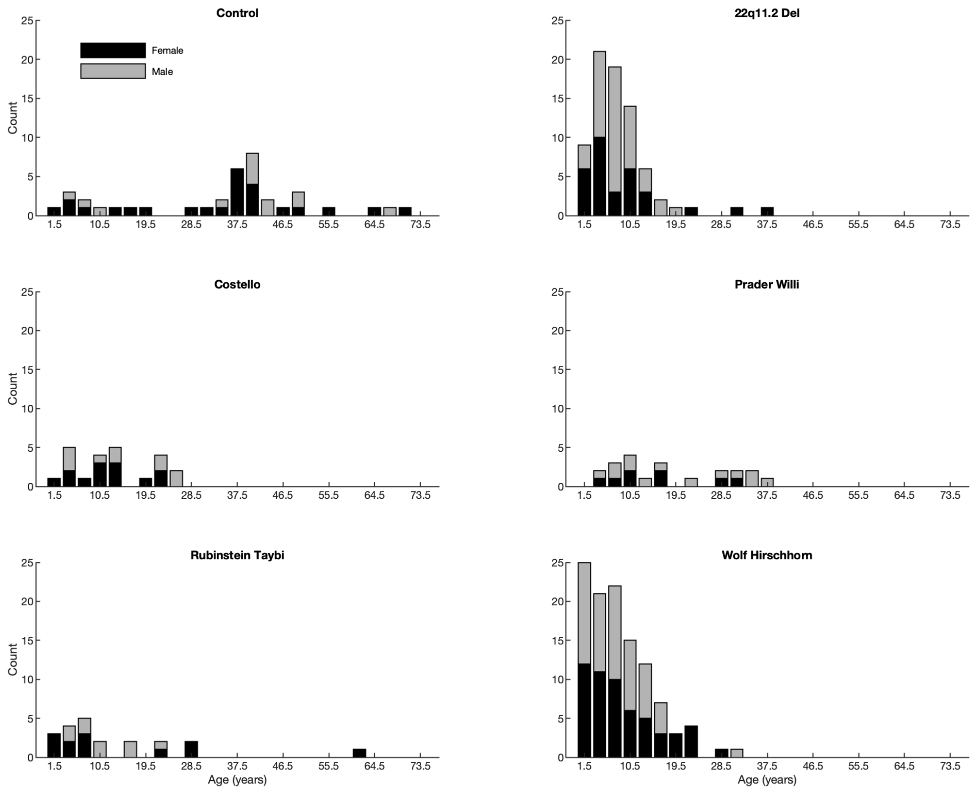

S1 Text Figure 2 Demographic characteristics of the test samples in all groups. This figure was generated in MATLAB 2021a (https://www.mathworks.com/).

1.3.2 Image processing and non-rigid registration

If necessary each image was cleaned to remove extra-facial areas that might make the registration difficult. Experience suggests that any hair on the forehead must be removed as well as structure close to the chin. E.g. if the face is nestled into the neck or if the face is very obese so that the fat of the neck renders the chin ill-defined, then the neck needs to be carefully removed. Five landmarks were roughly positioned on the inner corners of the eyes, the outer corners of the mouth and the tip of the nose on each facial image and on the template. A rigid Procrustes transformation calculated from the landmarks on the template to the landmarks on the target was applied to the template image to roughly align it to the target. This was followed by a rigid iterative closest point and non-rigid iterative closest point registration (as described in (1)), bringing the points of the template image onto the surface of the target image.

1.3.3 Visual assessment of registration failures

The anatomical correspondence induced by the registration was first visually assessed by viewing the original image of each individual, overlaid with the deformed template, with the anatomical positions (see above) emphasized as colored points. E.g. S1 Text Figure 3 shows the results of the mapping of an image of author HM.

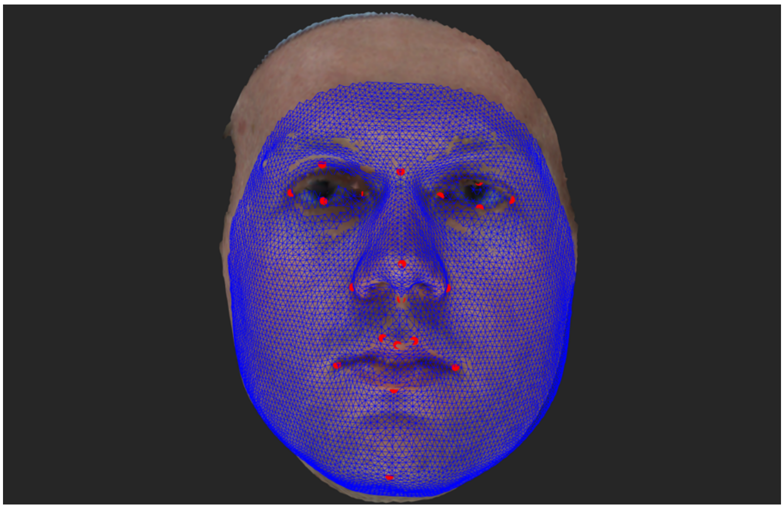

S1 Text Figure 3 Visualizing the anatomical correspondences. The deformed template image (blue wireframe) visualized in the same window as the original image. The points of the template that correspond to the anatomical loci are emphasized as red points. The accuracy of the correspondences can be assessed by comparing the position of these points with the corresponding anatomical locations on the target image. All points are quite accurately placed, although a slight error is visible on the left exocanthion. This figure was generated in MATLAB 2021a (https://www.mathworks.com/).

Registration is considered to have failed when multiple landmarks are moved significantly from their true anatomical location. Small differences from perfect anatomical precision (as can be seen for the right palpebral superius in the figure above) are not classed as failures and are assessed in the next section.

1.3.4 Types of registration failures

The two most common types of registration failures are 1) that the lower lip of the template (as indicated by the position of the labiale inferius) is mapped to the vermillion or cutaneous upper lip of the target, or to the palate (which can occur if the mouth is open) and 2) that the eyes are mapped to the lower or upper contour of the orbits, or to the cheek. Two examples of each type are shown in S1 Text Figure 4.

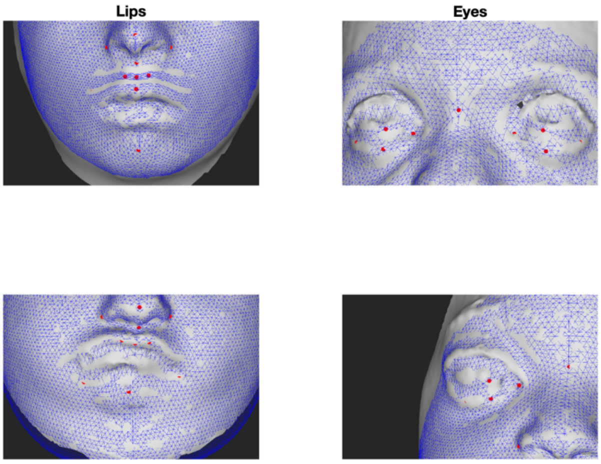

S1 Text Figure 4 Illustration of types of registration failures. This figure was generated in MATLAB 2021a (https://www.mathworks.com/).

1.3.5 Frequencies of registration failures

S1 Text Table 2 shows the registration success rate within each disease group (row) and S1 Text Table 3 shows the success rate for different ages. We noted 100% success rates for the faces of controls. In contrast, the more unusual facial shapes in the various disease groups lead to a lower success rate of ~87-100%. The success rates observed in all age groups are between 90-100%.

S1 Text Table 2 Success rate within each disease group as percentages and counts.

Group	Success rate
**Control**	100.0% (38/38)
**22q11.2 Del**	93.3% (70/75)
**Costello**	87.0% (20/23)
**Prader Willi**	100.0% (21/21)
**Rubinstein Taybi**	100.0% (21/21)
**Wolf Hirschhorn**	92.8% (103/111)

Total	94.5% (273/289)

S1 Text Table 3 Success rate within different age brackets as percentages and counts.

Age	Success rate
**0-2**	93.8% (15/16)
**2-4**	92.5% (37/40)
**4-6**	92.3% (36/39)
**6-8**	91.9% (34/37)
**8-10**	91.2% (31/34)
**10-12**	95.2% (20/21)
**12-14**	100.0% (19/19)
**14-16**	90.9% (10/11)
**16-18**	100.0% (10/10)
**18-75**	98.4% (61/62)
**Total**	94.5% (273/289)

1.3.6 Anatomical precision of the mapping

To quantitatively evaluate the precision of the correspondences induced by the registration the anatomical landmarks on the template were reconstructed on each registered face (as shown in S1 Text Figure 3). The Euclidean distance between these landmarks and the landmarks manually annotated on each face were calculated. S1 Text Table 4 plots the median and 5th and 95th percentiles of these distances per landmark for each disease group. S1 Text Table 5 plots the same statistics for different age groups.

S1 Text Table 4 Median (5th-95th percentiles) of the distance (mm) between the landmarks automatically placed by the non-rigid registration and the ground truth landmarks manually indicated onto each face by disease group.

	al_l	al_r	ch_l	ch_r	cp_l	cp_r	en_l	en_r	ex_l	ex_r
**Control**	1.56 (0.54-2.82)	0.99 (0.39-2.26)	1.21 (0.38-4.87)	1.19 (0.47-4.00)	1.42 (0.38-3.02)	1.38 (0.29-2.29)	1.58 (0.64-3.76)	1.57 (0.66-3.30)	1.67 (0.53-3.23)	1.83 (0.58-3.53)
**22q11_2 Del**	1.55 (0.55-2.83)	1.19 (0.41-2.90)	1.77 (0.34-9.16)	1.56 (0.30-7.16)	2.53 (0.62-4.88)	2.15 (0.54-5.01)	2.43 (1.08-4.17)	2.05 (0.86-3.78)	1.53 (0.43-3.63)	1.55 (0.64-3.84)
**Costello**	2.06 (1.04-3.79)	1.92 (0.75-4.05)	2.16 (0.32-10.25)	1.86 (0.59-9.94)	2.98 (0.82-7.82)	2.46 (1.15-7.36)	2.39 (0.74-7.25)	1.68 (0.81-6.77)	2.65 (0.90-7.58)	2.51 (0.68-7.03)
**Prader Willi**	2.92 (0.65-6.49)	2.25 (0.40-4.27)	1.82 (0.54-5.72)	1.76 (0.59-5.64)	1.78 (0.82-4.95)	1.52 (0.43-3.00)	2.68 (0.48-3.68)	1.76 (0.50-3.35)	1.88 (0.58-4.74)	1.89 (0.80-3.93)
**Rubinstein Taybi**	0.92 (0.45-2.45)	1.02 (0.30-2.30)	1.46 (0.28-4.20)	1.48 (0.59-5.81)	1.72 (0.43-3.81)	1.57 (0.27-3.06)	2.56 (0.70-4.06)	1.98 (0.51-3.11)	1.59 (0.57-6.16)	2.26 (0.63-6.57)
**Wolf Hirschhorn**	1.35 (0.50-3.08)	1.02 (0.25-2.28)	1.62 (0.47-5.54)	1.68 (0.50-5.73)	2.51 (1.07-6.14)	2.48 (0.91-5.45)	2.02 (0.68-6.78)	1.48 (0.63-4.37)	2.35 (0.67-7.88)	2.49 (0.90-7.15)
**Total**	1.50 (0.53-3.58)	1.14 (0.34-2.98)	1.65 (0.40-5.57)	1.58 (0.43-5.78)	2.33 (0.74-5.68)	2.06 (0.53-5.16)	2.28 (0.71-4.87)	1.76 (0.67-4.12)	1.84 (0.57-6.34)	2.01 (0.66-6.55)

	gn	li	ls	n	pi_l	pi_r	prn	ps_l	ps_r	sn
**Control**	2.66 (0.91-5.25)	1.50 (0.41-3.15)	0.88 (0.32-2.46)	1.85 (0.50-3.31)	1.52 (0.61-3.30)	1.49 (0.42-3.25)	0.77 (0.35-1.92)	1.16 (0.29-4.09)	1.64 (0.44-4.14)	0.91 (0.40-2.51)
**22q11_2 Del**	4.18 (1.76-7.41)	2.86 (1.07-15.21)	2.40 (0.61-4.76)	1.67 (0.76-4.01)	1.56 (0.53-3.57)	1.49 (0.58-3.43)	1.16 (0.28-2.20)	1.97 (0.44-4.97)	2.02 (0.74-4.86)	1.51 (0.43-3.56)
**Costello**	4.90 (1.94-14.62)	3.33 (0.87-23.04)	2.85 (0.45-9.54)	1.81 (0.43-4.03)	2.09 (0.64-10.67)	1.94 (0.68-10.65)	1.28 (0.24-3.02)	1.80 (0.49-9.42)	1.72 (0.60-8.96)	0.79 (0.40-2.41)
**Prader Willi**	9.19 (1.27-15.79)	2.75 (0.36-5.59)	1.70 (0.39-5.49)	1.75 (0.40-2.80)	2.01 (0.81-3.90)	1.90 (0.58-3.50)	1.15 (0.40-2.68)	2.22 (0.94-4.23)	1.72 (0.85-3.36)	2.78 (1.32-5.33)
**Rubinstein Taybi**	6.19 (2.17-11.08)	2.12 (0.35-4.99)	1.73 (0.63-3.98)	1.43 (0.22-3.71)	1.23 (0.42-4.65)	1.84 (0.43-4.53)	0.92 (0.33-1.66)	2.11 (0.53-5.68)	1.70 (0.49-3.99)	2.15 (0.81-3.56)
**Wolf Hirschhorn**	3.57 (0.96-8.20)	2.19 (0.62-4.98)	2.25 (0.90-5.29)	2.48 (0.61-6.16)	2.22 (0.46-8.13)	2.54 (0.65-6.61)	1.12 (0.29-2.46)	2.13 (0.59-8.42)	1.97 (0.47-7.46)	1.09 (0.27-3.16)
**Total**	3.98 (1.05-10.11)	2.28 (0.60-5.87)	2.06 (0.56-5.13)	1.92 (0.57-4.70)	1.84 (0.53-5.98)	1.90 (0.58-5.24)	1.08 (0.30-2.29)	1.93 (0.47-7.18)	1.93 (0.57-5.97)	1.30 (0.39-3.51)

S1 Text Table 5 Median (5th-95th percentiles) of the distance (mm) between the landmarks automatically placed by the non-rigid registration and the ground truth landmarks manually indicated onto each face by age group.

	al_l	al_r	ch_l	ch_r	cp_l	cp_r	en_l	en_r	ex_l	ex_r	gn	li	ls	n	pi_l	pi_r	prn	ps_l	ps_r	sn
**0-2**	1.47 (0.77-2.50)	1.04 (0.22-2.10)	1.44 (0.53-8.04)	1.31 (0.25-6.45)	2.77 (1.14-6.61)	2.07 (0.84-5.72)	2.38 (0.76-6.57)	1.85 (0.54-4.93)	1.87 (0.90-7.46)	1.95 (0.90-6.66)	5.24 (1.57-9.82)	1.32 (0.56-13.98)	2.08 (0.77-7.15)	1.86 (1.19-4.53)	2.22 (0.37-7.35)	2.22 (1.02-5.77)	1.15 (0.50-2.14)	1.40 (0.53-7.03)	2.34 (0.83-5.46)	1.10 (0.36-2.80)
**2-4**	1.42 (0.44-2.75)	0.97 (0.29-2.20)	1.57 (0.54-8.86)	1.63 (0.65-9.58)	2.43 (1.19-5.08)	2.10 (0.99-4.59)	2.95 (0.97-5.67)	2.11 (0.73-3.96)	1.79 (1.04-4.41)	2.01 (0.85-5.48)	4.36 (1.75-11.07)	2.06 (0.73-16.82)	2.16 (0.75-4.62)	2.28 (1.04-4.43)	1.77 (0.50-5.87)	1.70 (0.55-5.13)	1.09 (0.36-2.39)	2.19 (0.53-6.30)	2.58 (0.50-5.53)	1.26 (0.23-2.79)
**4-6**	1.50 (0.60-3.74)	1.14 (0.27-3.33)	1.61 (0.36-4.63)	1.27 (0.49-5.99)	2.49 (0.42-4.14)	2.26 (0.83-4.61)	2.35 (1.02-6.91)	1.79 (0.81-6.18)	1.97 (0.73-7.63)	2.06 (1.09-7.29)	4.43 (2.48-10.30)	2.72 (1.00-5.89)	2.40 (0.90-4.45)	2.06 (0.53-5.83)	1.82 (0.44-9.26)	1.58 (0.58-9.00)	1.16 (0.20-2.04)	2.22 (0.24-8.22)	2.02 (0.72-8.28)	1.24 (0.34-3.17)
**6-8**	1.48 (0.63-3.18)	1.11 (0.27-2.12)	1.71 (0.63-3.95)	1.70 (0.34-4.25)	1.98 (0.82-5.55)	2.06 (0.39-4.69)	2.54 (0.76-4.67)	2.08 (0.65-4.12)	1.55 (0.53-6.28)	1.53 (0.72-5.91)	4.39 (1.13-8.02)	2.35 (0.48-5.46)	2.06 (0.56-5.03)	1.87 (0.55-5.77)	2.20 (0.52-5.74)	1.98 (0.37-4.28)	1.25 (0.26-1.83)	1.78 (0.43-8.98)	1.62 (0.61-8.95)	1.35 (0.42-3.14)
**8-10**	1.27 (0.36-3.62)	1.15 (0.31-2.78)	1.96 (0.23-5.56)	1.85 (0.32-5.62)	2.49 (0.91-4.90)	2.27 (0.81-5.09)	2.16 (0.54-6.38)	1.80 (0.79-4.80)	1.93 (0.29-8.10)	1.91 (0.61-6.88)	3.51 (1.06-9.89)	2.35 (1.40-7.03)	2.32 (0.37-4.79)	1.79 (0.42-4.92)	1.47 (0.50-8.60)	1.68 (0.56-7.56)	0.95 (0.28-2.60)	1.78 (0.78-8.20)	1.77 (0.62-7.40)	1.91 (0.28-3.64)
**10-12**	1.48 (0.65-3.15)	1.35 (0.28-3.19)	1.81 (0.63-8.32)	1.53 (0.49-7.59)	2.66 (1.17-6.57)	2.65 (0.75-5.70)	2.10 (0.56-4.75)	1.60 (0.81-4.16)	1.82 (0.66-4.07)	1.69 (0.49-4.37)	3.86 (1.80-12.42)	2.64 (0.70-12.70)	2.31 (0.65-5.93)	2.34 (0.70-4.74)	1.70 (0.64-4.46)	1.90 (0.49-4.12)	0.95 (0.36-2.27)	2.22 (0.69-4.65)	1.69 (0.46-4.26)	0.96 (0.43-3.75)
**12-14**	1.46 (0.25-3.36)	1.26 (0.41-2.88)	1.77 (0.56-6.59)	1.16 (0.51-6.50)	2.67 (1.58-6.64)	2.57 (1.27-6.36)	1.90 (0.76-3.52)	1.48 (0.71-2.67)	1.75 (0.69-4.37)	2.19 (0.97-6.04)	3.67 (1.22-6.13)	2.91 (1.10-5.52)	3.07 (0.35-7.09)	2.00 (0.71-5.29)	1.71 (0.65-3.35)	1.92 (0.99-5.73)	1.17 (0.31-2.19)	1.92 (0.47-2.57)	1.38 (0.51-4.47)	1.26 (0.43-3.80)
**14-16**	2.08 (1.11-4.22)	1.34 (0.64-3.40)	2.16 (0.34-12.25)	2.44 (0.98-11.78)	2.05 (0.83-7.65)	1.57 (0.57-7.23)	2.34 (1.04-4.75)	1.56 (0.33-2.08)	1.68 (0.97-7.77)	3.03 (0.94-6.66)	5.84 (0.81-23.96)	2.60 (0.40-21.87)	2.06 (0.75-9.66)	1.39 (0.54-2.53)	2.39 (1.61-8.43)	2.54 (1.10-3.87)	1.12 (0.61-2.16)	2.71 (0.38-7.02)	1.62 (0.63-4.66)	1.31 (0.21-2.13)
**16-18**	1.74 (0.92-6.52)	1.16 (0.35-3.49)	1.58 (0.37-5.21)	1.69 (0.85-6.71)	2.48 (1.18-6.15)	2.05 (0.39-3.42)	2.16 (0.74-7.87)	2.01 (1.35-3.12)	1.44 (1.10-7.88)	1.53 (0.37-4.08)	3.88 (1.59-7.53)	2.99 (1.56-7.29)	2.08 (1.12-4.95)	2.19 (0.44-3.47)	1.82 (0.77-6.33)	2.46 (0.56-3.09)	1.06 (0.37-2.27)	1.31 (0.86-8.23)	2.12 (0.46-4.93)	2.24 (0.18-4.65)
**18-75**	1.65 (0.51-3.71)	1.15 (0.42-4.02)	1.56 (0.36-4.67)	1.42 (0.40-5.70)	1.51 (0.29-7.54)	1.56 (0.39-7.42)	1.48 (0.57-3.80)	1.41 (0.59-3.86)	1.89 (0.56-6.30)	2.21 (0.61-6.57)	3.39 (0.71-10.49)	1.66 (0.43-5.11)	1.18 (0.48-6.65)	1.83 (0.39-3.30)	1.96 (0.60-3.89)	1.87 (0.51-3.82)	0.95 (0.31-2.61)	1.76 (0.49-6.35)	1.79 (0.56-5.31)	1.27 (0.46-3.96)
**Total**	1.50 (0.53-3.58)	1.14 (0.34-2.98)	1.65 (0.40-5.57)	1.58 (0.43-5.78)	2.33 (0.74-5.68)	2.06 (0.53-5.16)	2.28 (0.71-4.87)	1.76 (0.67-4.12)	1.84 (0.57-6.34)	2.01 (0.66-6.55)	3.98 (1.05-10.11)	2.28 (0.60-5.87)	2.06 (0.56-5.13)	1.92 (0.57-4.70)	1.84 (0.53-5.98)	1.90 (0.58-5.24)	1.08 (0.30-2.29)	1.93 (0.47-7.18)	1.93 (0.57-5.97)	1.30 (0.39-3.51)

2 Constructing age appropriate models of normal variation

2.1 Constructing models of normal variation.

2.1.1 General

The 3D 'growth curves' comprise a series of age and sex-specific expected faces and models of the variation around this expected face. In general, we adopt an approach similar to the Nadaraya-Watson kernel regression, more informally known as a 'sliding window' approach. Essentially, each model is derived from the training data in a way that emphasizes the influence of cases closest in age while downplaying the influence of cases further away in age.

2.1.2 Computing observation weights

Each model is based on the training data in such a way that the observations most similar in age to the target age are given more weight and those further away are given less. For a particular target age a, within either sex group each observation's age x_i was converted into a z score according to a normal distribution centered on *a* with standard deviation *s*.

$$z_i = \frac{x_i - a}{s}$$

The weight for each observation w_i was then calculated by evaluating the standard normal probability density function at z_i:

$$w_i = \frac{1}{\sqrt{2\pi}} e^{\frac{-z_i^2}{2}}$$

In the foregoing calculations we included only cases with $-3<z_i<3$ for computational efficiency.

The term s is the only tunable parameter for the model. It controls the range of ages used to estimate the model. Those cases within +-1 s of a have the most influence and those further away have less. We address the setting of this parameter in section 2.2.

2.1.3 Computing the expected face

It is most common in morphometric analysis to define the expected shape of a population as the average shape. While the weighted average could be used for our purposes, it is undesirable as the average is biased when data are asymmetrically distributed about the target age. Here we adopt the same approach that we did in (2) and instead use a local linear regression to estimate the expected face. Firstly each face was scaled to a common size and co-aligned to the sample average by Generalized Procrustes Analysis. Each face was then represented by row vector containing the x, y and z co-ordinates for each point on the face. These were ordered so that the first three elements of each row were the co-ordinates of the first point followed by the co-ordinates of the second point and so on. The number of response variables was therefore 3k (k = number of points with each point being a three dimensional co-ordinate). The matrix of response variables (Y) in the regression was a vertical concatenation of these vectors. The matrix of predictor variables (X) was a single column containing the ages of each observation. X and Y were column centered on their respective weighted means ($\bar{x}$ and $\bar{y}$) and each row of each matrix was multiplied by the square root of their corresponding weight. PLSR was then performed without further column centering using the SIMPLS algorithm.[4]

This fitted the multivariate regression function is:

$$MX = \hat{Y}$$

Evaluating this function at $a - \bar{x}$ and adding $\bar{y}$ back onto the results produced co-ordinates of the expected face at age a.

2.1.4 Weighting, re-centering and statistical age adjustment

To ensure the variation is calculated relative to this expected face, all faces in the training sample were co-aligned to the expected face by scaled rigid Procrustes superimpositions. To exclude variation due to including cases of different ages in the estimate, we statistically adjust for this variation. Essentially this synthetically transforms all faces to the target age, and its purpose is to make the estimate of variation as reflective as possible of variation at the target age. This age adjustment is achieved by fitting a second weighted PLSR of these re-aligned shapes onto age. *Y* was this time column centered on the coordinates of the expected face and *X* was centered on the target age (*a*). The residuals of this regression model ϕ constitute weighted and age-adjusted displacements of the vertices of each face from the expected face.

2.1.5 Modelling normal variation

2.1.5.1 Pointwise standard deviations

To calculate the standard deviations at each point the 3D displacements away from the expected face in each row of ϕ were reshaped into a k (vertices) x 3 dimensions array. The displacements at each point was then projected separately onto each of the x, y and z axes (corresponding to the anatomical lateral-medial; superior-inferior; or anterior-posterior

directions), the direction perpendicular to the surface of the expected face, each giving a scalar displacement value d_j for each jth point. The total displacement (the Euclidean length of the 3D displacement vector) was also calculated. The standard deviation at the jth point in a given direction was then calculated:

$$\sigma_j = \sqrt{\frac{\sum_{i=1}^{k} d_{ij}^2}{\sum_{i=1}^{n} w_i}}$$

2.1.5.2 Statistical shape model

The modes of variation were calculated using a singular value decomposition of ϕ.

$$USV^T = \Phi$$

Where columns of V are the modes of variation. 'Scores' or 'projections' onto these modes of variation for each observation were calculated as:

$$A = \Phi V$$

The character A is an c (observations) × *l* (modes of variation) matrix. The variance in scores along the mth mode is calculated:

$$\delta_m^2 = \frac{\sum_{i=1}^{n} A_{i,m}{}^2}{\sum_{i=1}^{n} w_i}$$

Not all of the possible (n-1) modes of variation are meaningful. Therefore, each statistical shape model was trimmed to include only the modes of variation required to explain up to 98 percent of the variation. Specifically modes were added (l was increased) in order of decreasing variance until the following condition was satisfied:

$$\frac{\sum_{m=1}^{l} \delta_m^2}{\sum_{m=1}^{n-1} \delta_m^2} > .98$$

2.2 Age-adaptive bandwidth setting

For defining age and sex-appropriate models of normal facial variation, using the framework described above, there is only one parameter to be set: the bandwidth of the function, determining the weights (s). This should be big enough so as to incorporate sufficient training observations to model the true variation at that age (avoiding over-fitting to the training data). However, it should also be kept as minimal as possible to reduce the potential of under-fitting. In the extreme case, under-fitting would result in identical models being produced for every age.

The degree to which a statistical shape model (SSM) represents the variation in the population can be operationalized as the degree to which individuals not used to train the model can be represented as a linear combination of the modes of variation. This is termed 'generalization'. The degree to which an individual can be represented on the modes of a given SSM can be expressed numerically by their reconstruction error (see section 4.4).

To tune the bandwidth of the weighting function we first model how SSM generalization changes as a function of age and bandwidth: each individual in the normative training data aged less than 70 years was held out of the training data in turn, and an age and sex appropriate model was built from the remaining data. The reconstruction error of the held-out observation with respect to this model was calculated. This was repeated using 12 bandwidths linearly spaced from 1-12 years inclusive. Below S1 Text Figure 5 illustrates how reconstruction error changes as a function of age and bandwidth. The top rows scatter

reconstruction error against age and bandwidth and shows a surface fitted to this scatter. The bottom row shows a height map of this fitted surface where color is indexed to the reconstruction error. To give some context to these values Booth et al (3) built an extremely comprehensive single SSM of the human face from 10 000 scans, achieving an overall mean reconstruction error value of ~0.3mm. In general, generalization decreases (reconstruction error increases), especially for males, in the older ages. This is likely the combined product of two factors: 1. The training data are relatively sparsely sampled at these ages and 2. variation may have less structure at these ages and require more training data in general to model it.

The fluctuation in generalization suggests that no single bandwidth will be appropriate for all ages. Therefore, we allow the bandwidth to change depending on the particular age being modelled. Specifically, in the plot below we identified the isoline corresponding to a reconstruction error of 0.6mm. Following this isoline along the x-axis gives, for each age, the appropriate bandwidth to use to achieve a reconstruction error of 0.6mm. For each age, these are the bandwidths that we use. Beyond the age of 52 for males we cannot achieve the desired amount of generalization. We therefore truncate the growth curve at age 52.

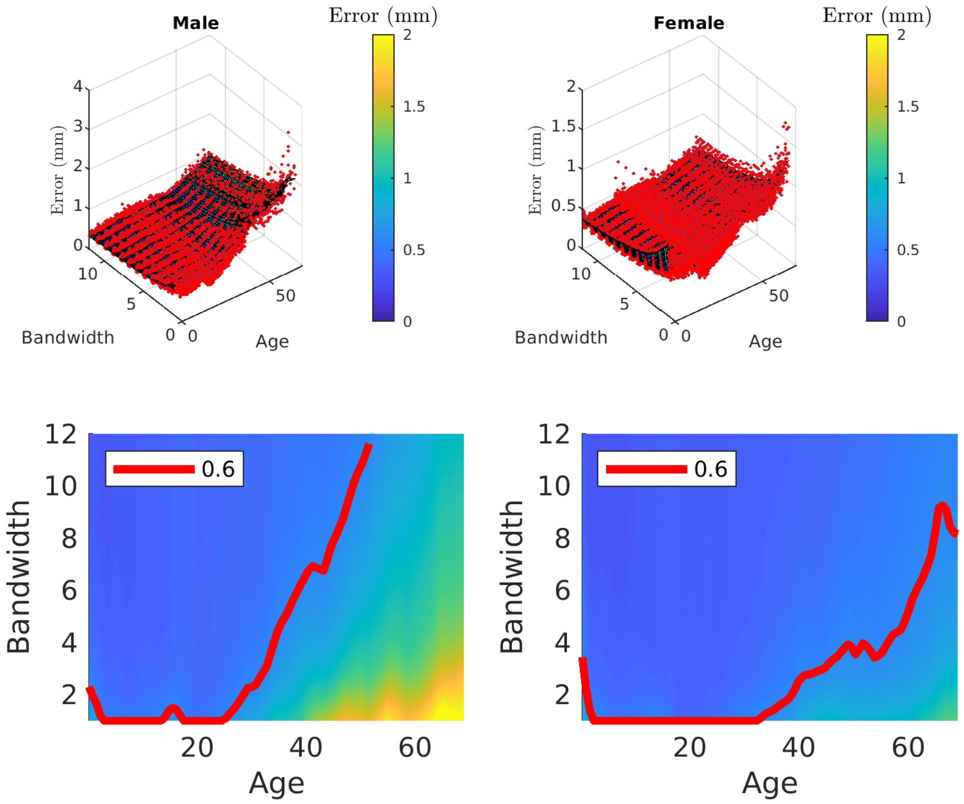

S1 Text Figure 5 Reconstruction error as a function of age and bandwidth. Top row shows the scatter of reconstruction errors, age and bandwidth. The fitted surface is a linear radial basis function interpolant fitted to the scattered data with smoothing constant set to 10, using MATLAB's 'rbfcreate' function. The bottom row shows a height map of the fitted surface applying the same color-map. The red line shows the isoline corresponding to a reconstruction error of 0.6mm and plots how the bandwidth of the models was changed as a function of age. This figure was generated in MATLAB 2021a (https://www.mathworks.com/).

3 Patient Assessment from models of normal facial variation

3.1 Patient assessment using age-appropriate models

3.1.1 Facial Signatures

Facial signatures are calculated by first aligning the face to the expected face of their age and sex appropriate model, using a robust Procrustes transformation. The robust Procrustes algorithm iteratively defines 'outlier weights' for each point on the face based on the

distances between the points of the two configurations (section 4.1) and computing a Procrustes transformation weighted so as to increase the influence of those points that are most similar, relative to those that are most different (section 4.2). The purpose of this is to reduce the ‘pinnochio effect’ in superimposition whereby large and localized differences between two configurations have a deleterious effect on the superimposition (4).

The z score of each point on the face is calculated by dividing the displacement of the face from the expected face along the desired dimension (x, y, z, normal or total) by the corresponding standard deviation of that point.

3.1.2 Normal equivalent

The normal equivalent algorithm aims to determine scores on the modes of variation of a statistical shape model that best represent the individual in question, while remaining within the bounds of normal facial variation. It comprises four steps that are iterated through until convergence. On the first iteration the normal equivalent face is initialized with the expected face of the statistical shape model and the outlier weights are initialized with ones.

3.1.2.1 Step 1: Alignment to the model

The face is aligned to the normal equivalent face using a weighted Procrustes transformation (section 4.2).

3.1.2.2 Step 2: Outlier weights

Outlier weights between the patient and the normal equivalent face are estimated for each point on the face, detecting the regions that are ‘abnormal’ (section 4.1). Abnormal regions

are those points on the patient that are relatively poorly coded by the statistical shape model (i.e. those that are most different from the normal equivalent).

3.1.2.3 Step 3: Weighted constrained projection of the patient onto the model

In theory any 'normal' face from the modelled population can be reconstructed as a series of transformations along each mode of variation away from the expected face. More formally a face can be represented as series of scores (α) that are the coefficients of a linear combination of the modes of variation. In this algorithm scores are calculated via a weighted projection onto the modes of variation (section 4.3) which emphasizes the reconstruction of 'normal' regions with high weights and de-emphasizes the reconstruction of regions with low weights, essentially interpolating the regions of low weights based on the regions of high weights.

To ensure this reconstruction lies within the normal range of variation α we make use of the Mahalanobis distance. Assuming multivariate normality, the squared Mahalanobis distance follows a chi-squared distribution with degrees of freedom equal to the number of modes of variation. This means that α can be constrained on statistical grounds with reference to a p value. By default we use a p-value of.05 although this can be altered by the user of the toolbox. Simply, we need to find the critical value of the Mahalanobis distance for a given p value and degrees of freedom. This is the square root of the critical value of chi-squared. We then calculate the Mahalanobis distance of α from the origin (ξ).

$$\xi = \sqrt{\sum_{m=1}^{l} \left(\frac{\alpha_m}{\delta_m}\right)^2}$$

Where δ_m is the square root of the variance along the mth mode. If ξ is greater than the critical value, α is scaled so that its Mahalanobis distance is equal to the critical value. This is done by multiplying each element in α by the ratio of the critical value of the Mahalanobis distance to ξ.

4 Further technical details

4.1 Calculating outlier weights

Outlier weights are calculated for each point on the face. They are based on the distribution of distances between two configurations of corresponding points P and Q and their value sharply decreases as the distance exceeds some user-defined threshold to classify the point as an outlier.

First, we to calculate the distances (d) for each corresponding point between P and Q:

$$d_j = \sqrt{\left(P_{1,j} - Q_{1,j}\right)^2 + \left(P_{2,j} - Q_{2,j}\right)^2 + \left(\left(P_{3,j} - Q_{3,j}\right)^2\right)}$$

Then each distance is converted into a z score according to a distribution with a mean of zero and a standard deviation of σ:

$$z_j = \frac{d_j}{\sigma}$$

where σ is:

$$\sigma = \sqrt{\frac{\sum_{j=1}^{k}(d_j^2 o_j)}{\sum_{j=1}^{k} o_j}}$$

where o is a vector of outlier weights (usually from a previous iteration within an iterative algorithm or all set to one). Each z score is then plugged into the equation of the standard normal probability density function to give the vector of probabilities b.

$$b_j = \frac{1}{\sqrt{2\pi}} e^{\frac{-z_i^2}{2}}$$

The outlier weights o are then calculated:

$$o_j = \frac{b_j}{b_j - \lambda}$$

where the value of λ is equal to:

$$\lambda = \frac{1}{\sqrt{2\pi}} e^{\frac{-k_i^2}{2}}$$

and serves to modify the weights according to the user-defined cut-off κ. The value of κ is in standard deviations. In this work we set κ=2.

4.2 Weighted Procrustes transformation

In this section we describe how to calculate the weighted Procrustes transformation. The influence of each point on the head depends on its outlier weight o_j.

The two landmark configurations will be denoted by P and Q. Both are 3 x k (points on the face) arrays. The translation of P to Q is coded in the difference between their weighted means: $\bar{p}$ and $\bar{q}$ respectively, where (for example) $\bar{q}$ is calculated:

$$\left[\frac{\sum_{j=1}^{k} Q_{1,j}}{\sum_{j=1}^{k} o_j}, \frac{\sum_{j=1}^{k} Q_{2,j}}{\sum_{j=1}^{k} o_j}, \frac{\sum_{i=1}^{k} Q_{3,j}}{\sum_{j=1}^{k} o_j}\right]$$

The scaling factor to scale *P* to the size of *Q* is the ratio of the size of *P* to the size of *Q*. The size of each configuration is measured as the mean Euclidean distance of all points from their weighted means.

Rotation in three dimensions is coded by a 3x3 rotation matrix. This is calculated by first centring *P* and *Q* on their weighted means, and then scaling each configuration so that the mean distance of all their points from the origin is equal to one. The weighted rotation can then be calculated by first performing a singular value decomposition:

$$USV^T = QOP^T$$

where *O* is a diagonal matrix with the outlier weights along its main diagonal *diag(o),* then calculating a 3 x 3 rotation matrix *H*:

$$H = VU^T$$

4.3 Weighted projection onto the statistical shape model.

The weighted projection onto the modes of variation of the statistical shape model is accomplished as follows. First the 3k (number of co-ordinates of all k points in all 3 dimensions) x l (modes of variation) in V modes of variation are each scaled according to their variances and the elements of each mode are scaled according to their outlier weights.

$$G = OVW$$

Where W is an l x l diagonal matrix containing the variances along each mode of variation and O is a 3k x 3k diagonal matrix containing outlier weights corresponding the elements in the columns of V $[o_1 \quad o_1 \quad o_1 \ldots o_k \quad o_k \quad o_k]$.

The singular value decomposition of this matrix gives:

$$U\mathrm{E}R^T = G$$

The projections onto the modes of variation are then:

$$\alpha = WR\Gamma U^T Ox$$

Where x is a column vector the co-ordinates (after subtracting the co-ordinates of the expected face that is the model center) of the observation to be projected onto the modes. The character Γ is an l x l diagonal matrix with elements along the main diagonal equal to:

$$\omega_m = \frac{\epsilon_m}{\epsilon_m^2 + \eta}$$

Where $\epsilon_m$is the mth singular value pulled from the main diagonal of E. The character η is an additional user-defined regularization factor. In previous work this has been used to constrain the normal equivalent to statistically plausible solutions (5). We employ a different approach to this constraint (see section 3.1.2.3). So η was always set to zero.

4.4 Computing reconstruction error

To calculate this for an individual face it was translated, rotated and scaled to the expected face of the given model (as described in section 4.2, applying all equal outlier weights), then their scores on the SSM were computed:

$$\alpha = xV$$

Where x is a 1 x 3k (k being the number of points on the face) vector containing co-ordinates of the face. V is a 3k x l (modes of variation) matrix of the modes of variation. The character α is a 1 x l vector of scores on each mode of variation. This operation is equivalent to the weighted projection (section 4.3) with equal weights and $\eta = 0$.

The face was reconstructed:

$$\hat{x} = V\alpha^T$$

Then the co-ordinates of the expected face were added back on to $\hat{x}$. The resulting co-ordinates were then scaled rotated and translated back into the co-ordinate system of the of the original shape by inverting the Procrustes transformation estimated at the beginning of this section. Then distance between corresponding points was calculated. The reconstruction error is the mean of these distances.

5 References

1. White JD, Ortega-Castrillón A, Matthews H, Zaidi AA, Ekrami O, Snyders J, et al. MeshMonk: Open-source large-scale intensive 3D phenotyping. Sci Rep. 2019 Apr 15;9(1):6085.

2. Matthews H, Penington A, Hardiman R, Fan Y, Clement J, Kilpatrick N, et al. Modelling 3D craniofacial growth trajectories for population comparison and classification illustrated using sex-differences. Sci Rep. 2018;8.

3. Booth J, Roussos A, Zafeiriou S, Ponniahy A, Dunaway D. A 3D morphable model learnt from 10,000 faces. In: 2016 IEEE Conference on Computer Vision and Pattern Recognition (CVPR). Las Vegas, NV, USA: IEEE; 2016. p. 5543–52.

4. Claes P, Daniels K, Walters M, Clement J, Vandermuelen D, Suetens P. Dysmorphometrics: The modelling of morphological abnormality. Theor Biol Med Model. 2012;9.

5. Claes P, Walters M, Gillett D, Vandermeulen D, Clement J, Suetens P. The normal-equivalent: a patient-specific assessment of facial harmony. Int J Oral Maxillofac Surg. 2013;42(9):1150–8.
